# Membrane Fusion Mediated by Non-covalent Binding of Re-engineered Cholera Toxin Assemblies to Glycolipids

**DOI:** 10.1021/acssynbio.2c00266

**Published:** 2022-11-11

**Authors:** Sarah Wehrum, Lina Siukstaite, Daniel J. Williamson, Thomas R. Branson, Taras Sych, Josef Madl, Gemma C. Wildsmith, Wenyue Dai, Erik Kempmann, James F. Ross, Maren Thomsen, Michael E. Webb, Winfried Römer, W. Bruce Turnbull

**Affiliations:** † Faculty of Biology, Albert-Ludwigs-University Freiburg, Schänzlestraße 1, 79104 Freiburg, Germany; ‡ Bioss-Centre for Biological Signalling Studies, Albert-Ludwigs-University Freiburg, Schänzlestraße 18, 79104 Freiburg, Germany; § School of Chemistry and Astbury Centre for Structural Molecular Biology, University of Leeds, LS2 9JT Leeds, U.K..; ∥ Freiburg Center for Interactive Materials and Bioinspired Technology (FIT), Albert-Ludwigs-University Freiburg, Georges-Köhler-Allee 105, 79110 Freiburg, Germany; ⊥ Science for Life Laboratory, Department of Women’s and Children’s Health, Karolinska Institutet, 17165 Solna, Sweden; # School of Biomedical Sciences and Astbury Centre for Structural Molecular Biology, University of Leeds, LS2 9JT Leeds, U.K..

**Keywords:** lectins, synthetic glycobiology, protein engineering, giant unilamellar vesicles

## Abstract

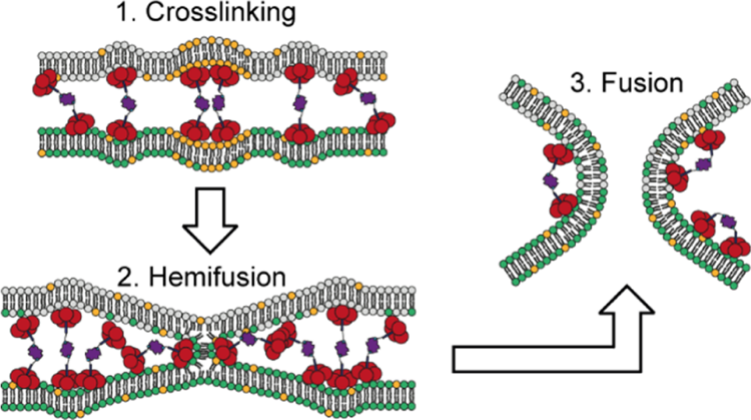

Membrane fusion is essential for the transport of macromolecules and viruses across membranes. While glycan-binding proteins (lectins) often initiate cellular adhesion, subsequent fusion events require additional protein machinery. No mechanism for membrane fusion arising from simply a protein binding to membrane glycolipids has been described thus far. Herein, we report that a biotinylated protein derived from cholera toxin becomes a fusogenic lectin upon cross-linking with streptavidin. This novel reengineered protein brings about hemifusion and fusion of vesicles as demonstrated by mixing of fluorescently labeled lipids between vesicles as well as content mixing of liposomes filled with fluorescently labeled dextran. Exclusion of the complex at vesicle–vesicle interfaces could also be observed, indicating the formation of hemifusion diaphragms. Discovery of this fusogenic lectin complex demonstrates that new emergent properties can arise from simple changes in protein architecture and provides insights into new mechanisms of lipid-driven fusion.

## Introduction

Confinement is fundamentally important for living systems, allowing the segregation of different biochemical environments through the use of lipid bilayers. In order to maintain the integrity of the boundaries of cells or organelles, many cellular processes require membrane fusion to transport impermeable macromolecules between compartments through the exchange of trafficking vesicles.^1−4^ Among the most intensively studied fusion proteins are the soluble *N*-ethylmaleimide-sensitive factor attachment protein receptor (SNARE) proteins. When a four-helix coiled-coil bundle is formed between SNARE proteins of opposing membranes, sufficient free energy is released to pull the membranes together and induce fusion.^3−5^ Furthermore, extra- and intracellular fusion of pathogens with host cells is essential for infectivity. For example, enveloped viruses use transmembrane glycoproteins with short amphiphilic peptide domains to insert into the target membrane. A subsequent conformational change brings the viral and target membrane into close proximity to enable fusion.^1,6,7^

The mechanisms of this transition from adhesion to fusion remain a matter of ongoing investigation but are best described by the stalk hypothesis.^1,8,9^ In this model, fusion proceeds through a stalk intermediate in which the outer (proximal) but not the inner (distal) membrane leaflets of approaching membranes have fused. Expansion of the fusion stalk results in a hemifusion diaphragm (HD) until formation of a fusion pore in the HD completes the fusion reaction. Many details of the underlying molecular mechanisms have been addressed using simpler lipidated model systems^10^ including small molecules,^11^ coiled-coil structures,^12−14^ or complementary DNA^15−17^ or peptide nucleic acid strands.^18^

**Figure 1:**
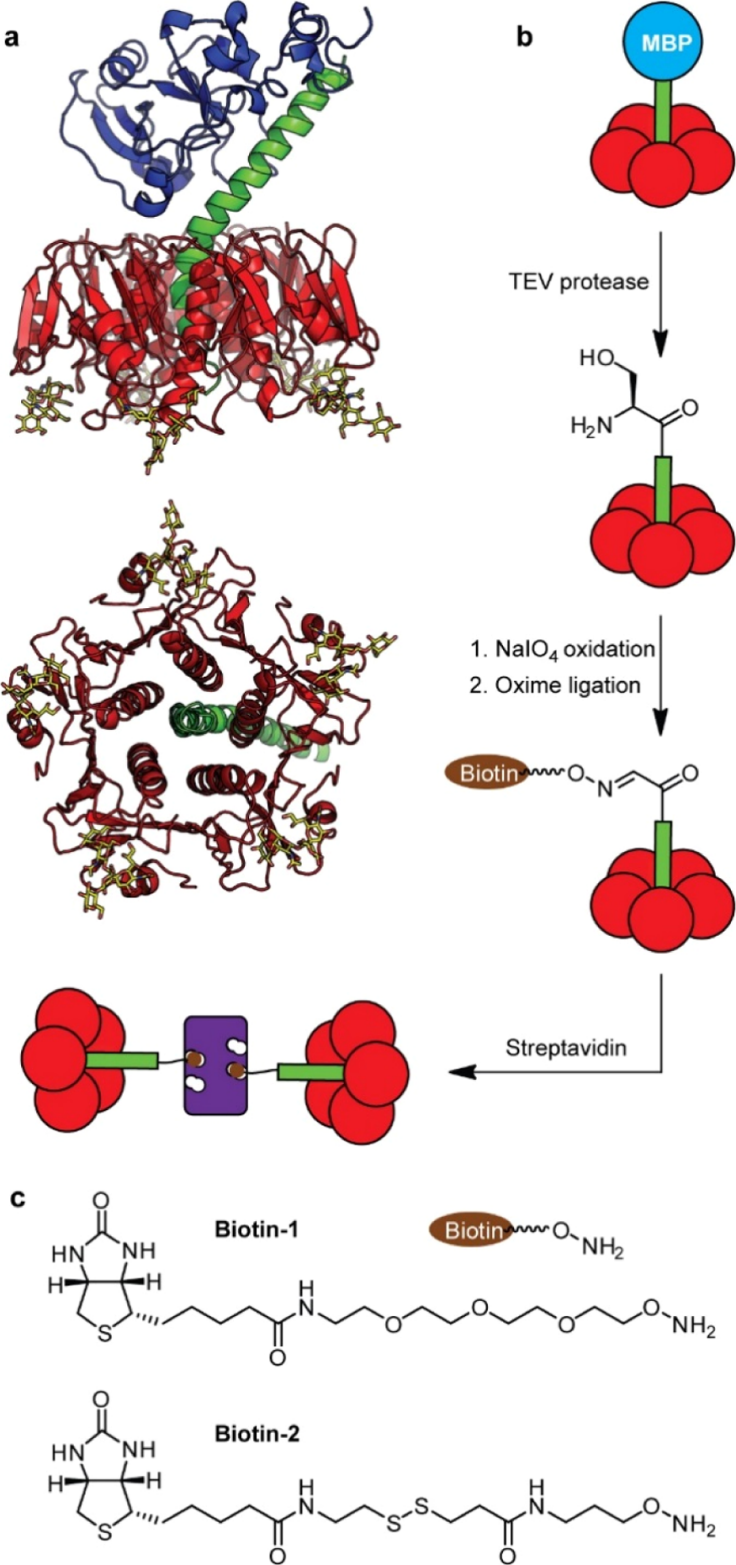
(a) Model of cholera toxin based on a superposition of protein data bank files 3CHB and 1XTC showing the toxic A1-subunit (blue), the A2-linker peptide (green), the pentameric B-subunit (CTB; red), and its carbohydrate ligand GM1 ganglioside (yellow); (b) a maltose-binding protein (MBP) fusion to the A2-peptide is cleaved using TEV protease to leave a serine residue that can be oxidized for oxime ligation to (c) oxyamine-biotin derivatives, prior to cross-linking with streptavidin.

While some proteins are inherently fusogenic, other proteins, such as bacterial toxins, can mediate adhesion and membrane bending reminiscent of the early stages of endocytosis^19^ but lack the ability to fuse membranes together. For example, cholera toxin from *Vibrio cholerae* is a member of a larger family of AB_5_ toxins which comprise a single toxic A-subunit associated with a non-toxic, pentameric B-subunit (CTB).^20^ The A-subunit is composed of the enzymatically active A1-domain and the A2-domain which protrudes through the central pore of the donut-shaped ring formed by the CTB pentamer (Figure 1a).^21^ The latter is responsible for the initial adhesion to enable the toxin to enter host cells by specific binding to the high-affinity ligand (*K*_d_ = 10–40 nM for a monovalent interaction) GM1 ganglioside through its branched pentasaccharide.^22^ The crystal structure of CTB reveals one binding site for GM1 per protomer, all of which are on the same face of the pentamer.^23^ Therefore, the lectin is pre-disposed to bind to several ligands on a single membrane, which leads to receptor clustering,^24^ that under certain conditions can induce phase separation of membranes^25^ or the formation of tubular membrane invaginations, a prerequisite for endocytosis, as previously described for different AB_5_ toxins.^26−29^

In this study, we sought to investigate if a simple change in lectin architecture could give rise to new emergent properties in addition to, or in place of, the membrane invagination observed upon binding to cells and GM1-functionalized synthetic membranes.^29^ Therefore, a strategy was developed to prepare complexes of CTB pentamers, in which a non-toxic AB_5_ protein is modified with biotin by oxime ligation at the N-terminus of the A2 peptide chain to allow complexation with streptavidin in a controlled orientation (Figure 1b). We demonstrate that such novel biotin-modified AB_5_ cholera toxin–streptavidin complexes [Strep–(AB_5_)_*n*_] exhibit both cross-linking and fusogenic functions. In contrast to the aforementioned fusogenic strategies in which complementary recognition elements are lipidated and introduced into separate vesicles,^10−18^ the Strep–(AB_5_)_*n*_ is a soluble protein; that is, it is neither embedded nor covalently attached to the membrane but binds non-covalently to liposomes containing the GM1 ganglioside. Streptavidin-mediated cross-linking of two or more AB_5_ complexes allows the assemblies to bind to two opposing membranes in parallel, which is the first prerequisite for fusion. Hemifusion and fusion events were observed by transfer of fluorescently labeled lipids from one membrane to the other and mixing of the vesicles’ fluorescent content, respectively, and also leakage or rupture of liposomes. This emergent behavior is dependent upon the formation of the multimeric Strep–(AB_5_)_*n*_, as the parent CTB pentamer only induces membrane invaginations.^29^

## Results

### Preparation and Characterization of Biotinylated AB_5_ Complexes

**Figure 2:**
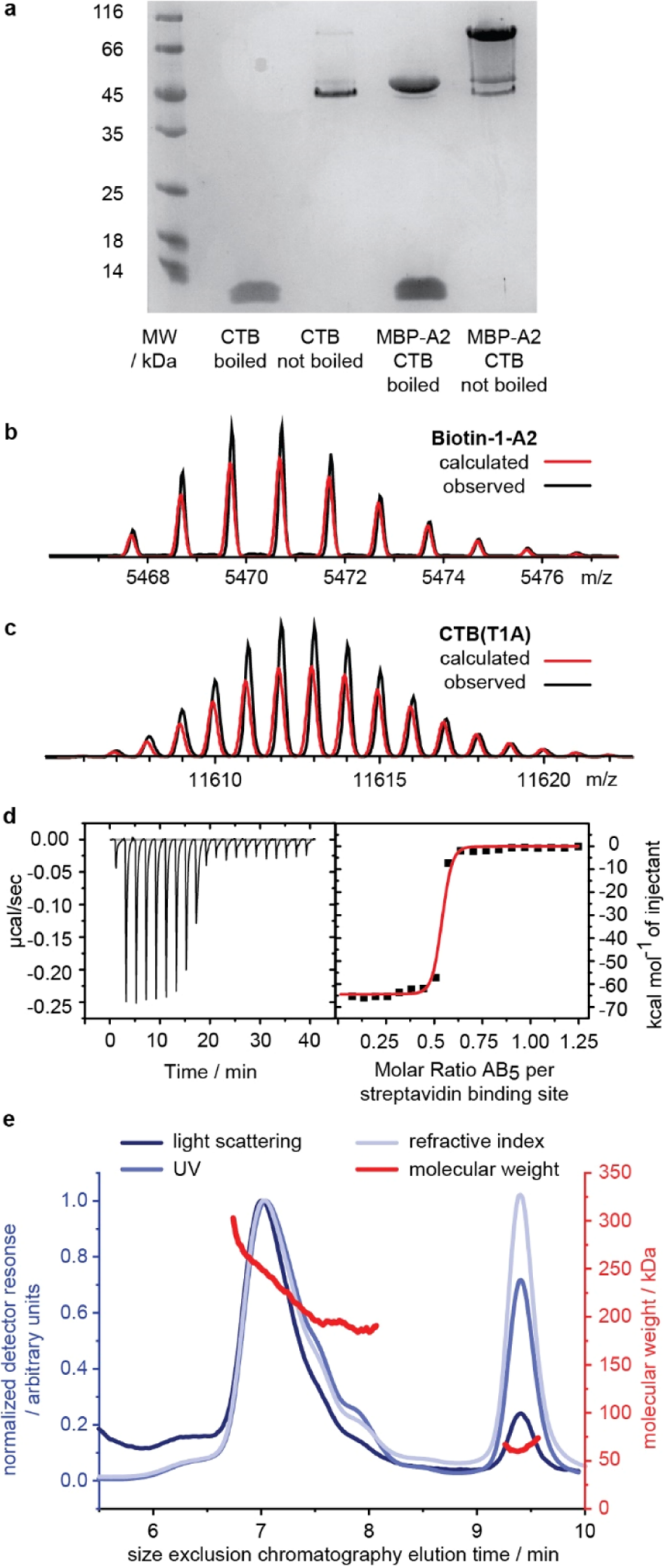
Preparation of biotinylated AB5 complex and its interaction with streptavidin. (a) SDS-PAGE comparison of CTB and an MBP-A2/CTB complex expressed from pSAB2.1: CTB migrates as a pentamer with an apparent molecular weight of ca. 45 kDa unless the sample is boiled prior to SDS-PAGE, in which case it migrates as an 11.5 kDa monomer. Boiling the AB_5_ complex also results in its dissociation into MBP-A2 and the CTB monomer, whereas the unboiled sample migrates as an AB_5_ complex, albeit with some dissociation into the MBP-A2 and CTB pentamers; theoretical calculated mass spectra (red) and observed deconvoluted spectra (black) for (b) A2-peptide modified with biotin-1 and (c) CTB protein; (d) isothermal titration calorimetry thermogram (left) and binding isotherm (right) showing that only 50% of streptavidin binding sites are accessible to the biotinylated AB_5_ complex; (e) SEC-MALS analysis of streptavidin–AB_5_ complexes. The graph shows the normalized response (left *y*-axis) of light scattering, refractive index, and UV absorbance detectors (blue lines) as a function of time (*x*-axis), following injection of the streptavidin–AB_5_ complexes on to the size exclusion column. The molecular weight in kDa (right *y*-axis) of species eluting at selected time points is depicted in red and indicates that assemblies with two to three AB_5_ complexes per streptavidin eluted between 6.5 and 8 min, while unbound AB_5_ protein eluted between 9 and 10 min.

A construct for periplasmic assembly of non-toxic AB_5_ analogues of cholera toxin in *Escherichia coli* cells was designed in analogy to earlier work by Jobling and Holmes.^30^ Plasmid pSAB2.1 (Supporting Information, Figure S7) allowed the co-expression of CTB and a maltose-binding protein (MBP)-A2 fusion protein to enable a two-step purification strategy for isolating the AB_5_ proteins. MBP-containing proteins could first be isolated using an amylose affinity resin, before removing wild-type *E. coli* MBP from the AB_5_ species by size exclusion chromatography (SEC), or by exploiting the inherent ability of CTB to bind to a nickel chelation resin.^31^ The resulting MBP-A2/CTB AB_5_ proteins were sufficiently stable to be observed by sodium dodecyl sulphate-polyacrylamide gel electrophoresis (SDS-PAGE) as long as the samples were not boiled prior to electrophoresis (Figure 2a). However, extended exposure of samples to the SDS-containing loading buffer prior to analysis led to some dissociation of AB_5_ species into their MBP-A2 and CTB pentamer components.

A tobacco etch virus (TEV) protease recognition site between the MBP and A2 domains was included to allow subsequent removal of the MBP domain to give an A2/CTB AB_5_ protein. The construct used for the oxime ligation strategy was designed to leave an N-terminal serine residue after cleavage with TEV protease (Figure 1b). It was therefore necessary to employ a threonine-to-alanine mutation at the N-terminus of the CTB sequence to prevent concomitant oxidation of the CTB protomers when exposed to periodate.^32,33^ Following TEV protease treatment, the A2/CTB AB_5_ species was repurified on a nickel-chelation resin and oxidized with sodium periodate. After 5 min, electrospray mass spectrometry (ES-MS) confirmed complete oxidation of the terminal serine group, which was subjected to aniline-catalyzed oxime ligation with the oxyamine-biotin derivative biotin-1 (Figure 1c). ES-MS analysis confirmed the formation of the biotinylated A2 peptide and also that CTB had been unaffected by the oxidation and oximation reactions (Figure 2b,c). Similar ES-MS results (Supporting Information, Figure S11) were obtained when disulfide-linked oxyamine biotin-2 (Figure 1c) was ligated onto the oxidized AB_5_ protein.

Complexation of the biotin-1–AB_5_ protein with streptavidin was studied using isothermal titration calorimetry (ITC) and size exclusion chromatography with multiple-angle light scattering (SEC-MALS) analysis. Titration of biotin-1–AB_5_ into a solution of streptavidin (protomer concentration) indicated that the titration was complete once half of the streptavidin binding sites were filled (Figure 2d). The binding sites of the streptavidin tetramer are arranged in pairs on opposite faces of the protein, with adjacent sites separated by ca. 20 Å. We thus presume that once one site is filled, the bulky protein appendage precludes easy binding to the adjacent site, and so the second biotinylated AB_5_ protein preferentially binds to the opposite face of the streptavidin.^34^ Analysis of a mixture of biotinylated AB_5_ and streptavidin by SEC-MALS indicated that the complexes had masses in the range of ca. 190–250 kDa, which is consistent with 2–3 AB_5_ proteins per streptavidin tetramer (Figure 2e). We would reconcile these observations by proposing that the 2:1 complexation observed by ITC initially dominates upon mixing the species, but higher complexes may also form over longer time periods in the presence of excess AB_5_ protein. For simplicity, we depict 2:1 complexes in Figures 1 and 6, but we note that the 3:1 complexes present will also contribute to the observed phenomena.

**Figure 3:**
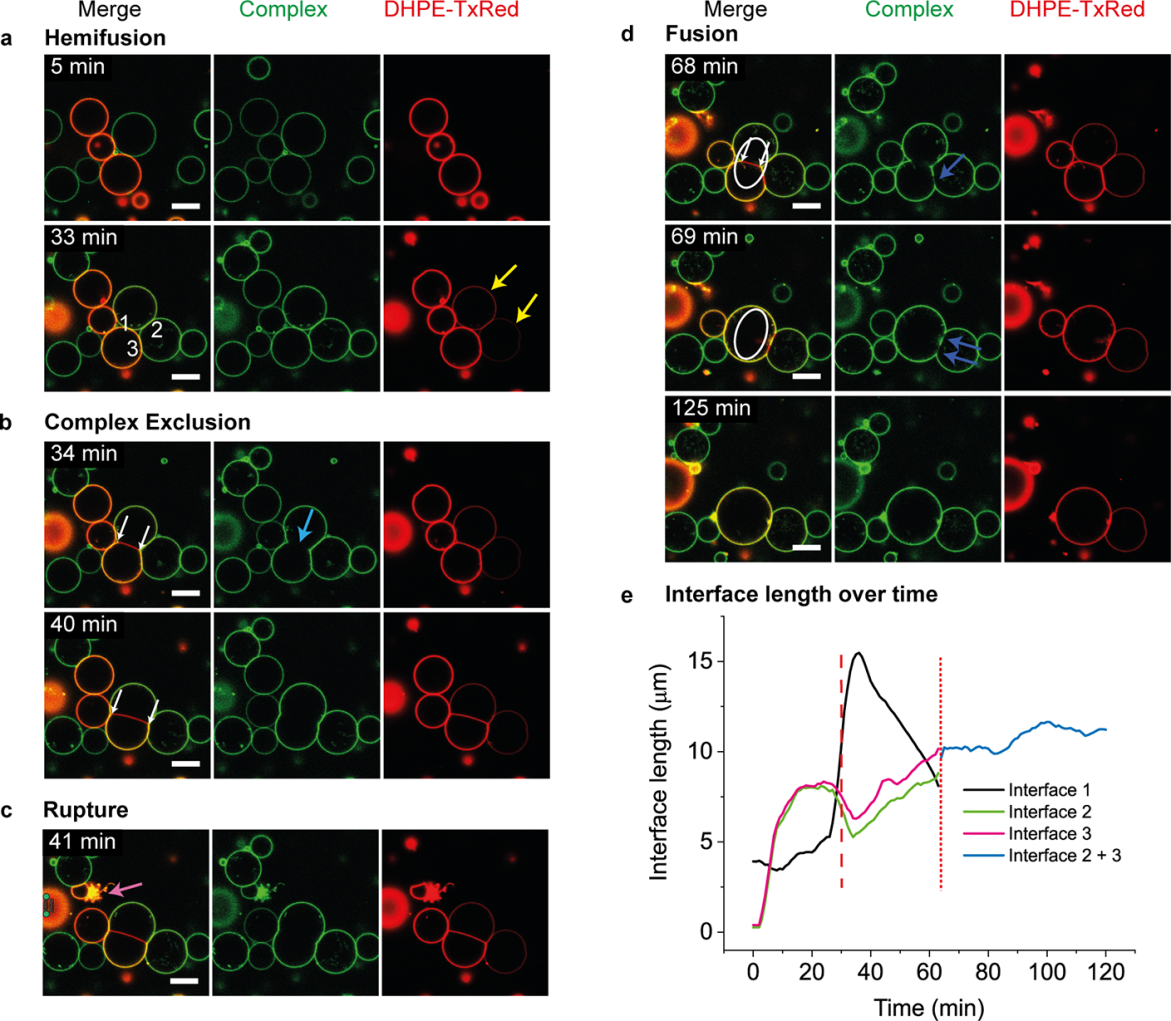
Hemifusion, fusion, or vesicle rupture can be induced by Strep–(AB_5_)_*n*_ time series captured in 1 min intervals of two slightly deflated vesicle populations containing 2.5 mol % GM1 and either no membrane dye or 0.5 mol % DHPE-TxRed (red) incubated with 100 nM AB_5_ complex [AB_5_–biotin −streptavidin–AF488 (green)] for indicated time periods. (a + b) Transfer of fluorescently labeled lipids (yellow arrows) to vesicles without membrane staining indicated hemifusion of the outer membrane leaflets. (b) Cross-linking of two vesicles resulted in an elongated interface which did grow in size (distance between white arrows), yet the AB_5_ complex itself was excluded from the contact site (blue arrow). (c) Vesicle rupture (pink arrow) was another frequent observation. (d) Two vesicles, which had already undergone hemifusion, then fused into one (indicated by a circle). Dark blue arrows point to domains within the interface of which the AB_5_ complex was excluded. (e) Dynamics of interface growth. Lengths of interfaces are derived from 2D images of the GUVs’ cross sections in the focal plane displayed in the figure. Interfaces corresponding to the data presented in the graph are highlighted in panel (a) by white numbers 1, 2, and 3. The dashed line marks the time point when interface 1 starts to transform into a HD. The dotted line indicates the point of fusion, after which interfaces 2 and 3 effectively become a single interface (displayed as “interface 2 + 3”). Scale bar is 10 μm. The full time series can be seen in the Supporting Information, Video SV3.

**Figure 4:**
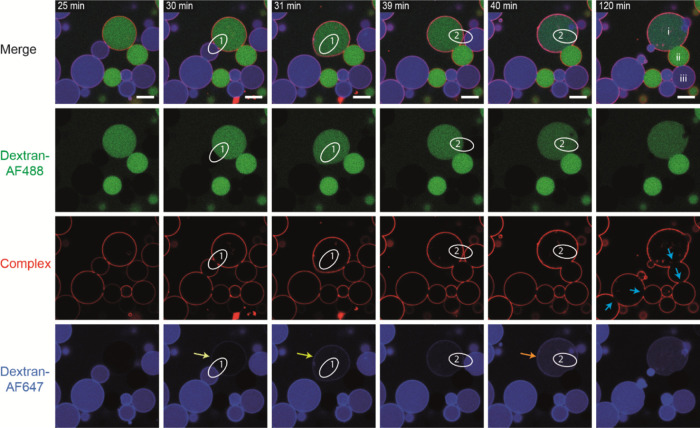
Complete fusion but not cross-linking results in content mixing of vesicle incubated Strep–(AB_5_)_*n*_ time series captured in 1 min intervals of two slightly deflated vesicle populations (5 mol % GM1 without membrane staining) filled with either dextran-AF488 (green) or dextran-647 (blue) incubated with 200 nM AB_5_–biotin–streptavidin–AF555 (red) complex. 30 min after AB_5_ complex incubation, a slight increase of blue fluorescence was observed for an otherwise green fluorescent vesicle (light yellow arrow), which could indicate the opening of a fusion pore. Indeed, only 1 min later, two vesicles fused into one, and the blue fluorescence intensity did further increase (yellow arrow). At 40 min, the resulting vesicle of fusion event 1 fused a second time with another dextran-647-containing vesicle, which further accelerated the blue fluorescence (orange arrow). Other vesicles showing significant cross-linking, even with the exclusion of Strep–(AB_5_)_*n*_ (blue arrows), did not show any signs of fusion or the formation of fusion pores, as contents remained completely separate (e.g., vesicle i, ii, and iii). Scale bar is 10 μm. The full time series can be seen in the Supporting Information, Video SV4.

**Figure 5:**
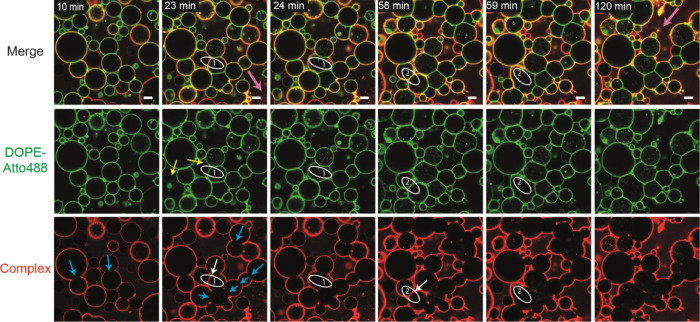
Vesicle fusion does not necessarily follow extensive vesicle cross-linking with complex exclusion. Time series captured in 1 min intervals of two slightly deflated vesicle populations containing 5 mol % GM1 and either no membrane dye or 0.5 mol % DOPE-Atto488 (green) incubated with 200 nM AB_5_–biotin–streptavidin–AF555 (red) complex for indicated time periods. Blue arrows indicate the appearance of first interfaces where the AB_5_ complex became excluded. White arrows, on the other hand, point at contact areas, where no exclusion was observed, but these vesicles did fuse within the next minute (first event at 24 min, second event at 59 min after Strep–(AB_5_)_*n*_ addition). Yellow arrows indicate the transfer of membrane dye indicating hemifusion. The pink arrows at 23 and 120 min are pointing at vesicles, which did rupture toward in the course of the time lapse. Scale bar is 10 μm. The full time series can be seen in the Supporting Information, Video SV5.

### Streptavidin–AB_5_ Complexes Cross-Link GM1-Coated Vesicles

The effect of the biotinylated AB_5_ proteins on membranes, both before and after complexation with streptavidin, was studied under well-defined conditions using giant unilamellar vesicles (GUVs). Vesicles with a lipid composition giving a liquid disordered (L_d_) phase (Supporting Information, Materials and Methods) and a defined amount of the GM1 ganglioside receptor, were mixed with the AB_5_ proteins/streptavidin complexes and observed by confocal microscopy. In the absence of streptavidin, incubation of biotinylated AB_5_ protein with vesicles resulted in the formation of tubular membrane invaginations (Supporting Information, Video SV1) in accordance with previous studies using CTB.^29^ In contrast to this behavior, when vesicles containing 1 mol % GM1 were incubated with 120 nM fluorescently labeled Strep–(AB_5_)_*n*_, they started to adhere to each other, giving rise to elongated, planar interfaces between vesicles (Supporting Information, Figure S1). In some cases, tubule formation could still be observed at locations on the GUVs that were distant from the cross-linked interfaces (Supporting Information, Video SV2). These observations were in line with our expectations that back-to-back assembly of AB_5_ proteins in the streptavidin complex should lead to vesicle cross-linking, as previously described by us for other carbohydrate-binding proteins that have binding sites that point in opposing directions.^35^

Vesicle cross-linking was dependent on complexation of the biotinylated AB_5_ protein with streptavidin. Fluorescently labeled streptavidin complexed with AB_5_ proteins bearing either a PEG-linked biotin-1 or disulfide-linked biotin-2 each cross-linked GM1-functionalized vesicles (Figure 1c; Supporting Information, Figure S2). However, incubation of the disulfide-linked complex with the reducing agent dithiothreitol (DTT) resulted in a decrease (6 h treatment, Supporting Information, Figure S2) or complete loss (27 h treatment) of both fluorescence and cross-linking, indicating that the fluorescently labeled streptavidin–biotin complex was released from the AB_5_ protein (albeit slowly, presumably resulting from slow diffusion of the fluorescent streptavidin protein from confinement at the interface of the cross-linked vesicles). Equivalent DTT treatment of vesicles cross-linked with Strep–(AB_5_)_*n*_ based on PEG-linked biotin-1 did not decrease cross-linking (Supporting Information, Figure S2).

### Streptavidin–AB_5_ Complexes Induce Intermediate States That Proceed to Fusion

In addition to cross-linking, other diverse effects were also observed, particularly at higher concentrations (2.5 or 5 mol %) of the ganglioside. Observation of a mixture of fluorescently labeled and unlabeled vesicles with 100 nM Strep–(AB_5_)_*n*_ over time (Figure 3 and Supporting Information, Video SV3) revealed several additional phenomena that can be interpreted as intermediates of membrane fusion. First, transfer of the membrane marker, the red-fluorescent phospholipid DHPE-TxRed, from one vesicle to an unlabeled vesicle indicated the occurrence of hemifusion of the outer membrane leaflets of approaching membranes (Figure 3a, yellow arrows).

Furthermore, Strep–(AB_5_)_*n*_ was often excluded from the contact site between vesicles (Figure 3b, blue arrow) or from small areas within interfaces (Figure 3d, dark blue arrows). This is an intriguing observation, as for other lectins with opposing binding sites, a strong accumulation within the interface was observed.^35^ The latter observation was attributed to restricted movement of the lectin within the planar interfaces that connect the vesicles. In contrast to the rather slow depletion of fluorescent streptavidin, when the disulfide-linked complex was treated with DTT (Supporting Information, Figure S2), initial exclusion of Strep–(AB_5_)_*n*_ in Figure 3b appeared suddenly between the 33 and 34 min frames. The planar interface continued to increase in size (Figure 3b, white arrows) without accumulation of the fluorescent complex between the vesicles. We propose that hemifusion of the outer leaflets indicates the formation of a fusion stalk that can expand into a HD in which the intact inner monolayers of each vesicle, which have not been exposed to the complex, make contact to form a single hybrid bilayer between the vesicles.

Other frequent observations included the rupture of vesicles with complete membrane failure (Figure 3c and Supporting Information, Video SV7) and liposome leakage without membrane lysis, indicated by a slow loss of the vesicle’s fluorescent dextran content (Supporting Information, Figure S3). Yet the most outstanding observation was the fusion of two vesicles into one (Figure 3d, white circle). In this case, the fusion was preceded by a reduction of the size of the putative HD contact area from which Strep–(AB_5_)_*n*_ was excluded (Figure 3d, white arrows). Dynamic changes in the size of interfaces between the fusing vesicles and a neighboring vesicle (interfaces 1–3 highlighted in Figure 3a) are illustrated in Figure 3e. While interfaces are two-dimensional structures, they are usually not arranged parallel to the focal plane; therefore, they appear as one-dimensional structures (lines) in the fluorescence images. We thus used the length of the apparent interface as a measure for interface size, as described in the Supporting Information.^36^ The data show an initial rapid increase in the length of interface 1 upon formation of the HD and then continuous contraction to the point of fusion (dotted line). In contrast, the lengths of interfaces 2 and 3 first decrease and then increase during this same period. This dynamic relationship of the size of HD interfaces between neighboring vesicles probably underpins the lack of correlation between interface size and GUV radius (Supporting Information, Figure S4). It is also worth noting that GUVs can rearrange in 3D, which might also affect the apparent interface length.

The fusion phenomenon could also be observed by mixing of vesicle contents employing GUVs containing 5 mol % GM1 and 200 nM Strep–(AB_5_)_*n*_ (Figure 4 and Supporting Information, Video SV4). Fusion of vesicles containing dextran-AF488 with vesicles containing dextran-AF647 resulted in vesicles containing both contents (Figure 4, yellow arrows). When a larger vesicle filled with dextran-AF488 fused with a smaller vesicle filled with dextran AF647, the fluorescence intensity of the latter became more diluted and showed a lower fluorescence intensity (Figure 4, 31 min), which then increased when a second dextran-AF647-filled vesicle subsequently fused (Figure 4, 40 min) with the latter. Interestingly, there was already a slight increase of AF647 fluorescence within the AF488 vesicle some time before fusion (Figure 4, 30 min), which could indicate that a small fusion pore had already formed. The accumulation of dextran-AF647 at the membrane can most likely be attributed to the properties of the fluorescent polysaccharide, as it was observed for all vesicles. Nonetheless, these experiments also provide proof that the membrane barrier was still intact for the majority of cross-linked vesicles that showed exclusion of Strep–(AB_5_)_*n*_ from the interface (Figure 4, blue arrows), as no mixing of those vesicles’ contents was observed (Figure 4, 120 min, vesicles i, ii, iii). This observation is also consistent with hemifusion, leading to formation of an HD between the vesicles.

We did not always observe exclusion of Strep–(AB_5_)_*n*_ from the interface between vesicles prior to fusion. While protein exclusion was observed in Figure 3 and fusion event 1 of Figure 4, there was no preceding exclusion of the complex observed in fusion event 2. Similarly, the two fusion events depicted in Figure 5 (Supporting Information, Video SV5) do not show elongated interfaces or complex exclusion before fusion. In this case, hemifusion could be observed for fusion event 1 by the transfer of the fluorescent phospholipid DOPE-Atto488 (Figure 5, yellow arrows). However, both vesicles involved in fusion event 2 contained the membrane dye, and accordingly, there could not be any indication of whether or not hemifusion did occur. It is possible that protein exclusion may have happened very briefly between consecutive frames of the video which were acquired at 1 min intervals. However, most interfaces of this time lapse that did show complex exclusion (Figure 5, blue arrows) were observable over an extended time, in many cases over 1 h, without proceeding to complete fusion.

**Figure 6:**
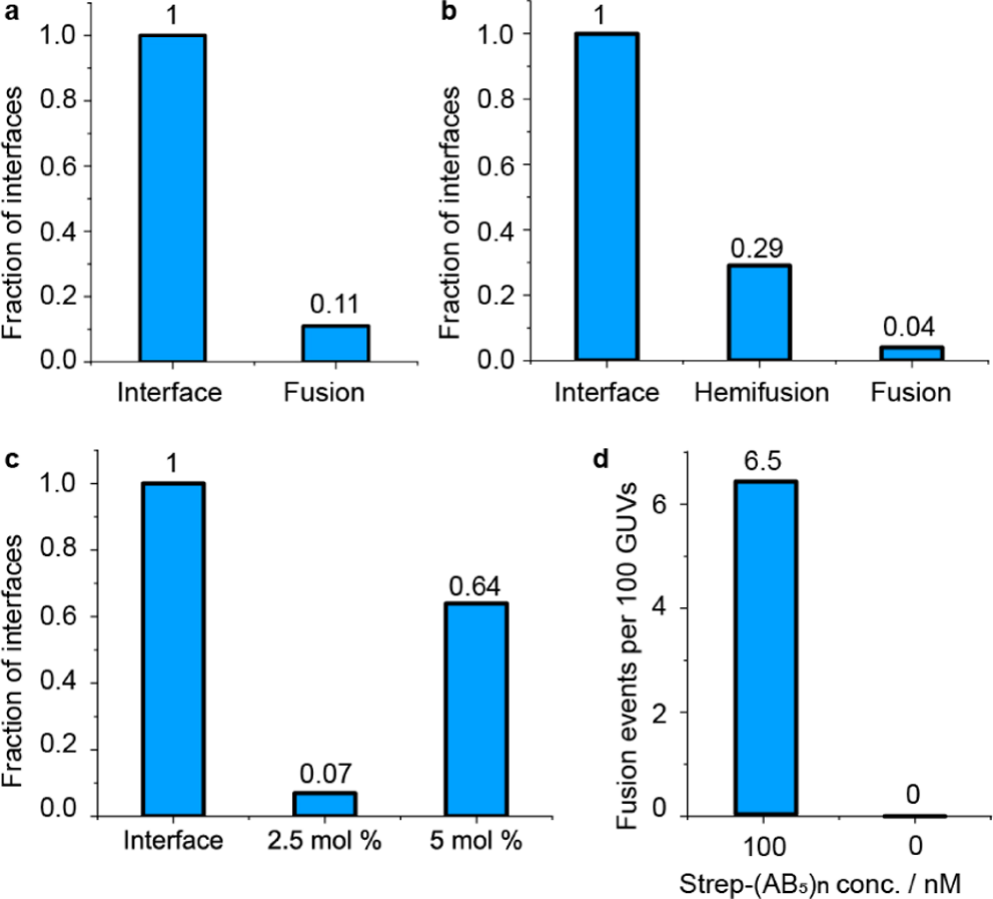
The fraction of the interfaces of the GUVs that undergo hemifusion or fusion events or form diaphragms. Data for panels (a,b) were extracted from the same data set comprising 349 GUVs (2.5% GM1; representative image: Figure 3). (a) 11% of the 205 GUV–GUV interfaces in this data set underwent fusion. (b) 98 of the interfaces in this data set were between labeled and unlabeled GUVs, 29% of which were observed to undergo hemifusion, and 13% of these (4% of the total) proceeded to full fusion. (c) Interfaces that transform into HDs for 2.5 and 5 mol % GM1 (representative images: Figures 3 and 5, respectively). (d) Fusion events observed per 100 vesicles in the presence (*n* = 349 GUVs) and absence (*n* = 195 GUVs) of 100 nM Strep–(AB_5_)_*n*_.

Data recorded under the conditions used for Figure 3 were used to quantify the fraction of GUV interfaces that undergo hemifusion and fusion, and data recorded for Figures 3 and 5 were used to quantify HD formation as a function of GM1 molar percentage (Figure 6). While interface size was found to be independent of GM1 concentration (Supporting Information, Figure S5), the number of interfaces per GUV increased with the percentage of GM1 (Supporting Information, Figure S6); therefore, for consistency, the data in Figure 6 are displayed as a fraction of interfaces rather than as a fraction of GUVs. A sample of 349 GUVs (comprising 2.5% GM1) formed 205 interfaces with their neighbors, and 11% of such interfaces underwent fusion (Figure 6a). Ninety-eight of these interfaces were between pairs of labeled and unlabeled vesicles, where it was also possible to quantify numbers of hemifusion events arising from transfer of labeled lipids (Figure 6b). Hemifusion was detected for 29% of interfaces between labeled and unlabeled GUVs, and 13% of these hemifused vesicles (i.e., 4% of the total) went on to undergo full fusion. In contrast, control experiments (e.g., Supporting Information, Video SV9) observing 195 GUVs (47% of which were labeled with the membrane dye) over the same time period (2 h) in the absence of Strep–(AB_5_)_*n*_ showed no evidence of hemifusion, fusion, or rupture. Overall, there were 6.5 fusion events per 100 vesicles in the presence of 100 nM Strep–(AB_5_)_*n*_ and no fusion events observed in the absence of the protein complex (Figure 6d). HD formation was found to be very dependent on GM1 concentration: only 7% of GUV interfaces underwent HD formation when 2.5% GM1 was used, but this rose to 64% of interfaces for GUVs with 5% GM1.

Variation in membrane elasticity, for example, arising from osmotic effects or lipid composition, also influences the outcome of the fusion experiments. For example, vesicle rupture was more frequently observed when the inner and outer solutions were osmotically matched (Supporting Information, Video SV7); on the other hand, slightly deflated GUVs (10 mOsm difference) were more likely to engage in hemifusion or fusion when incubated with Strep–(AB_5_)_*n*_ (Figures 3–5). In all experiments described thus far, the lipid composition was designed to give liquid disordered (L_d_) membranes. However, when 200 nM of Strep–(AB_5_)_*n*_ was applied to GUVs constituted of the lipid bilayer of the rigid liquid-ordered (L_o_) phase, tubule formation arising from negative membrane curvature was abolished. L_o_ GUVs still displayed cross-linking and HD formation, but no fusion or transfer of fluorescent lipid from labeled to unlabeled vesicles was observed during the 90 min timescale of the experiment (Supporting Information, Video SV8). Lipid diffusion is expected to be about 10-fold slower in L_o_ membranes,^37^ but the lack of labeled lipid transfer was perhaps surprising, as HD formation would be the result of hemifusion. However, it is notable that diffusion of fluorescent lipids between the hemifused vesicles in Figure 3 (Supporting Information, Video SV3) slows considerably upon formation of the HD. It is possible that the Strep–(AB_5_)_*n*_ complex bound at the edge of HD gives rise to a “restricted hemifusion” phenotype observed in other fusogenic systems,^38^ and this further restricts inter-vesicle lipid diffusion.

## Discussion

### Potential Mechanisms for CTB-Mediated Membrane Fusion

**Figure 7:**
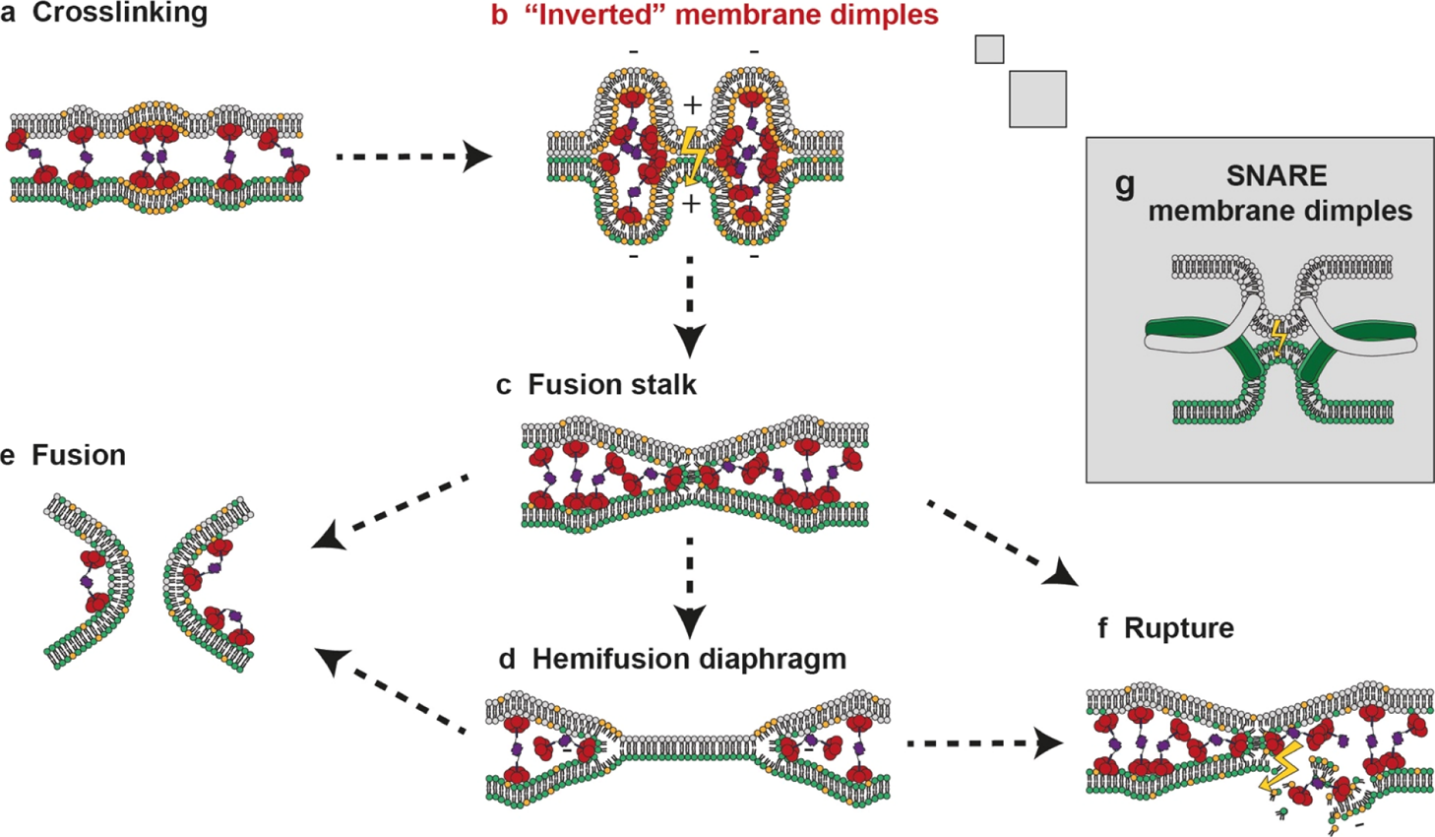
“Inverted” fusion stalk hypothesis. Schematic presentation of AB_5_ (red)–biotin–streptavidin (purple) complexes binding to lipid bilayers containing phospholipids (white lipids), GM1 gangliosides (orange lipids), and fluorescently labeled phospholipids (green lipids) describing the possible intermediates of membrane fusion. (a) In addition to membrane cross-linking, the complex could induce negative membrane curvature at multiple sites aligned on the opposing membranes possibly leading to (b) the formation of a cluster of multiple “inverted” membrane dimples with negative membrane curvature generating positive membrane dimples on opposing contacting membranes with high bending energies (illustrated as yellow lightning) in-between, which could be released by (c) the formation of a fusion stalk which could directly collapse into (e) a fusion pore or radially expand into (d) a HD from which the complex would become excluded. The negative membrane curvature at the HD rim could potentially be stabilized by Strep–(AB_5_)_*n*_ until (e) the opening of a fusion pore would complete fusion of the two vesicles. Alternatively, pore formation outside the fusion stalk or HD might result in (f) vesicle rupture or leakage. (g) Schematic presentation of the outward budded membrane dimples generated by SNARE-induced fusion.

Unlike other multimeric lectins, Strep–(AB_5_)_*n*_ is able to induce not just association but also fusion and rupture of GM1-functionalized vesicles. How then does this complex of glycan-binding proteins cause these observed phenomena? As briefly discussed in the introduction, the stalk model of membrane fusion describes the strong bending of two membranes into an hourglass shape in order to make a connection without the exposure of the hydrophobic lipid tails to water.^8,9^ Therefore, it has been proposed that a critical step of fusion is the local bending of membrane bilayers by fusion proteins into dimples that point toward the adjacent membrane (Figure 7g).^38^ Since such bending occurs at length scales below the diffraction limit for fluorescence microscopy, we cannot observe such changes in our data; however, it might be possible to observe such features using cryoelectron tomography.^39^ Nevertheless, it is well established that GM1 binding by CTB results in glycolipid clustering and leads to negative membrane curvature and invaginations (Figure 7a),^40^ in part as CTB applies a downward force to the membrane through protruding alpha helices in the middle of the protein.^41^ That is where we think the novel architecture of Strep–(AB_5_)_*n*_ comes into play: due to the linkage of two to three CTB molecules which are “pushing” into opposite directions, a cluster of several membrane dimples with negative curvature could give rise to positive membrane dimples in-between, which could align opposing membranes within contact distance (Figure 7b). Accordingly, the critical step of local bending still applies as strong bending energies that would build up at the rim of the inward bud, and the tip of the outside bud would be released by the formation of a fusion stalk (Figure 7c).

Formation of a fusion stalk accounts for the observed transfer of fluorescent lipids between adjacent vesicles and also provides an explanation for the frequently observed exclusion of Strep–(AB_5_)_*n*_ from the interface between vesicles. Radial expansion of the stalk would lead to a HD in which both inner membrane leaflets of the hemifused vesicles come together to create a single bilayer (Figure 7d). HDs in micrometer size have been observed previously in GUV systems^42^ and also during vacuolar lysosome fusion.^43^ It has been proposed that HD formation occurs in cases where tension of the outer leaflet of a vesicle is greater than that of the inner leaflet.^44^ Formation of a fusion stalk provides a mechanism to rebalance membrane tension by allowing retraction of the outer leaflet from the boundary between vesicles, which in turn leads to HD expansion.^44^ It is possible that Strep–(AB_5_)_*n*_ binding to GM1 may increase tension in the outer leaflet, for example, by ordering lipids,^45^ and release of this tension drives HD formation. This would be in line with our data in Figure 6c which shows that increasing the GM1 concentration, and thus GM1 clustering in the membrane, leads to a substantial increase in HD formation. It could potentially be possible to test the importance of GM1 clustering for membrane bending and HD formation by engineering AB_5_ complexes that have only one binding site and should thus not enable lipid clustering.^46^

An alternative strategy to test the importance of membrane bending is to use vesicles with a rigid liquid-ordered (L_o_) phase; however, switching from L_d_ to L_o_ vesicles did not prevent HD formation. It is known that L_o_ GUVs do not display the large tubular invaginations upon binding to CTB that are seen for L_d_ GUVs.^29^ However, that does not preclude the possibility that smaller-scale membrane bending could still occur for L_o_ GUVs, unobserved, below the diffraction limit of our fluorescence microscopy experiments. Indeed, membrane bending is presumably required for the hemifusion process leading to the HD. As full fusion was not observed in these experiments, L_o_-derived HDs appear to be more stable than those derived from L_d_ membranes, which is in line with other reports that L_o_ vesicles are less prone to membrane fusion.^47^

Irrespective of whether fusion occurs by direct collapse of the fusion stalk^38^ or rupture of the bilayer following the formation of an HD,^48,49^ the process necessitates the formation of a pore in the membrane (Figure 7e). Vesicle rupture and leakage also require pore formation but on a portion of the membrane that does not lie at the interface between two connected vesicles. We can only speculate on the forces introduced to the membrane by Strep–(AB_5_)_*n*_ that could cause membrane rupture. Simunovic and co-workers have recently presented a mechanism called friction-driven scission to describe how protein scaffolds can build local membrane tension until tubular membranes undergo scission through lysis when the tube gets elongated.^50^ The dynamic expansion and contraction of HDs seen in Figure 3e and Video SV3 indicate that there are significant forces acting on our GUVs, and any motions will presumably be subject to friction caused by inter-GUV cross-linking by Strep–(AB_5_)_*n*_. Thus, it is possible that observed membrane rupture may be subject to an analogous type of friction-driven scission. While it is beyond the scope of our present work to prove conclusively the precise mechanism by which fusion is induced by Strep–(AB_5_)_*n*_, our data provide a new perspective on the established fusion hypotheses and support a novel strategy for fusion, involving induction of multiple aligned membrane invaginations by a glycosphingolipid-binding protein.

## Conclusions

We have shown that site-specific biotinylation of an AB_5_ bacterial toxin permits the assembly of multimeric complexes with streptavidin which exhibit new fusogenic functions. While all five binding sites for the ganglioside GM1 of the CTB pentamer face in one direction, leading to receptor clustering and negative membrane curvature, the back-to-back assembly of the complex allows for additional cross-linking of two GM1-functionalized membranes resulting in vesicle clusters with planar interfaces. In contrast to previous studies in which avidin proteins have been used to cross-link biotinylated vesicles,^51,52^ the Strep–(AB_5_)_*n*_ complex was found to induce hemifusion indicated by fluorescently labeled lipid exchange between vesicles. While we have recently reported lipid mixing induced by a bifunctional “janus” lectin,^53^ which includes a *Ralstonia solanacearum* lectin domain that also has membrane-bending properties,^54^ the Strep–(AB_5_)_*n*_ complex can additionally fuse two vesicles into one with merging of the fluorescently labeled liposome contents. Exclusion of the Strep–(AB_5_)_*n*_ complex from the GUV–GUV interface was also frequently observed, indicating the formation of a HD. HD formation increased at higher concentrations of GM1. We propose that when clusters of Strep–(AB_5_)_*n*_ cross-link membranes, CTB-induced “inverted” membrane dimples, with very high bending energies, become aligned, and upon contact between opposing membranes, there is formation of a fusion stalk. Subsequent expansion of the stalk into a HD and opening of a fusion pore could complete the fusion process. This mechanism presents a different fusogenic strategy compared to the dimples budding out of a membrane by, for example, SNARE proteins or viral peptides that induce membrane fusion. This work has allowed us to identify a synthetic glycan-binding protein with fusogenic properties that acts by simply binding to a membrane component and does not require its own incorporation into the membrane. The results provide a new insight into the established hypotheses of membrane fusion, which is an indispensable requirement for many cellular processes as well as for applications like drug delivery.

## Supporting Information Available

Full experimental procedures for the preparation of proteins and protein conjugates; experimental procedures for isothermal titration calorimetry, SEC-MALLS, GUV preparation, and image analysis; and ^1^H and ^13^C NMR spectra for organic compounds and mass spectrometry data for modified proteins (PDF)
Supplementary videos of GUV experiments (ZIP)

## Notes

The raw data associated with this paper including fluorescence microscopy images, isothermal titration calorimetry, SEC-MALLS, NMR, and mass spectra are openly available from the University of Leeds data repository. https://doi.org/10.5518/1250.
