# Supplementary material for: Membrane Fusion Mediated by Non-covalent Binding of Re-engineered Cholera Toxin Assemblies to Glycolipids
Source: ACS Synth Biol. 2022 Nov 11;11(12):3929–38. doi: 10.1021/acssynbio.2c00266 (PMC9764410; doi:10.1021/acssynbio.2c00266)
Supplement: Supplementary file 1 — sb2c00266_si_001.pdf [file sb2c00266_si_001.pdf]

Membrane fusion mediated by non-covalent binding of re-engineered cholera toxin assemblies to glycolipids

Supporting Information

Sarah Wehrum‡[a,b], Lina Siukstaite‡[a,b], Daniel J. Williamson‡[c], Thomas R. Branson‡[c], Taras Sych[a,b,d,e], Josef Madl[a,b,d], Gemma C. Wildsmith[c], Wenyue Dai[c], Erik Kempmann[a,b], James F. Ross[c], Maren Thomsen[f], Michael E. Webb[c], Winfried Römer*[a,b,d] and W. Bruce Turnbull*[c]

[a.] *Faculty of Biology, Albert-Ludwigs-University Freiburg, Schänzlestraße 1, 79104 Freiburg, Germany.*

[b.] *Bioss - Centre for Biological Signalling Studies, Albert-Ludwigs-University Freiburg, Schänzlestraße 18, 79104 Freiburg, Germany.*

[c.] *School of Chemistry and Astbury Centre for Structural Molecular Biology, University of Leeds, Leeds LS2 9JT, UK.*

[d.] *Freiburg Center for Interactive Materials and Bioinspired Technology (FIT), Albert-Ludwigs-University Freiburg, Georges-Köhler-Allee 105, 79110 Freiburg, Germany*

[e.] *Science for Life Laboratory, Department of Women's and Children's Health, Karolinska Institutet, 17165, Solna, Sweden*

[f.] *School of Biomedical Sciences and Astbury Centre for Structural Molecular Biology, University of Leeds, Leeds LS2 9JT, UK*

‡These authors contributed equally.

Email w.b.turnbull@leeds.ac.uk, winfried.roemer@bioss.uni-freiburg.de

Contents

Supplementary Figures (S1-S6)	page 2
Supplementary video captions	page 7
Supplementary experimental methods	page 9
References	page 26

Supplementary Figures

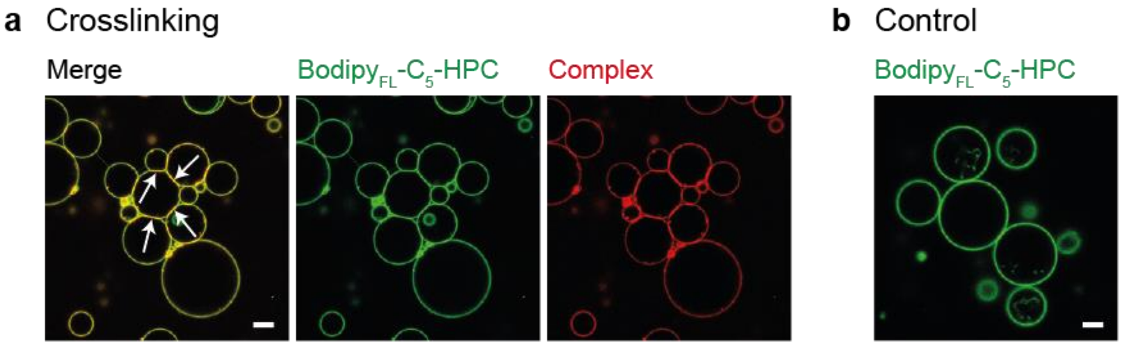

Supplementary Fig. S1: A simple change in architecture enables Strep-(AB_5)$_n$ to crosslink vesicles

Vesicles functionalized with 1 mol% GM1 and 0.5 mol% Bodipy$_{FL}$-C$_5$-HPC (green) were (**a**) crosslinked after 2 h with 120 nM AB_5-biotin – streptavidin-AF555 (red), indicated by elongated, planar interfaces (white arrows) while (**b**) vesicles without treatment showed minimal contact puncta. Scale bars are 10 µm.

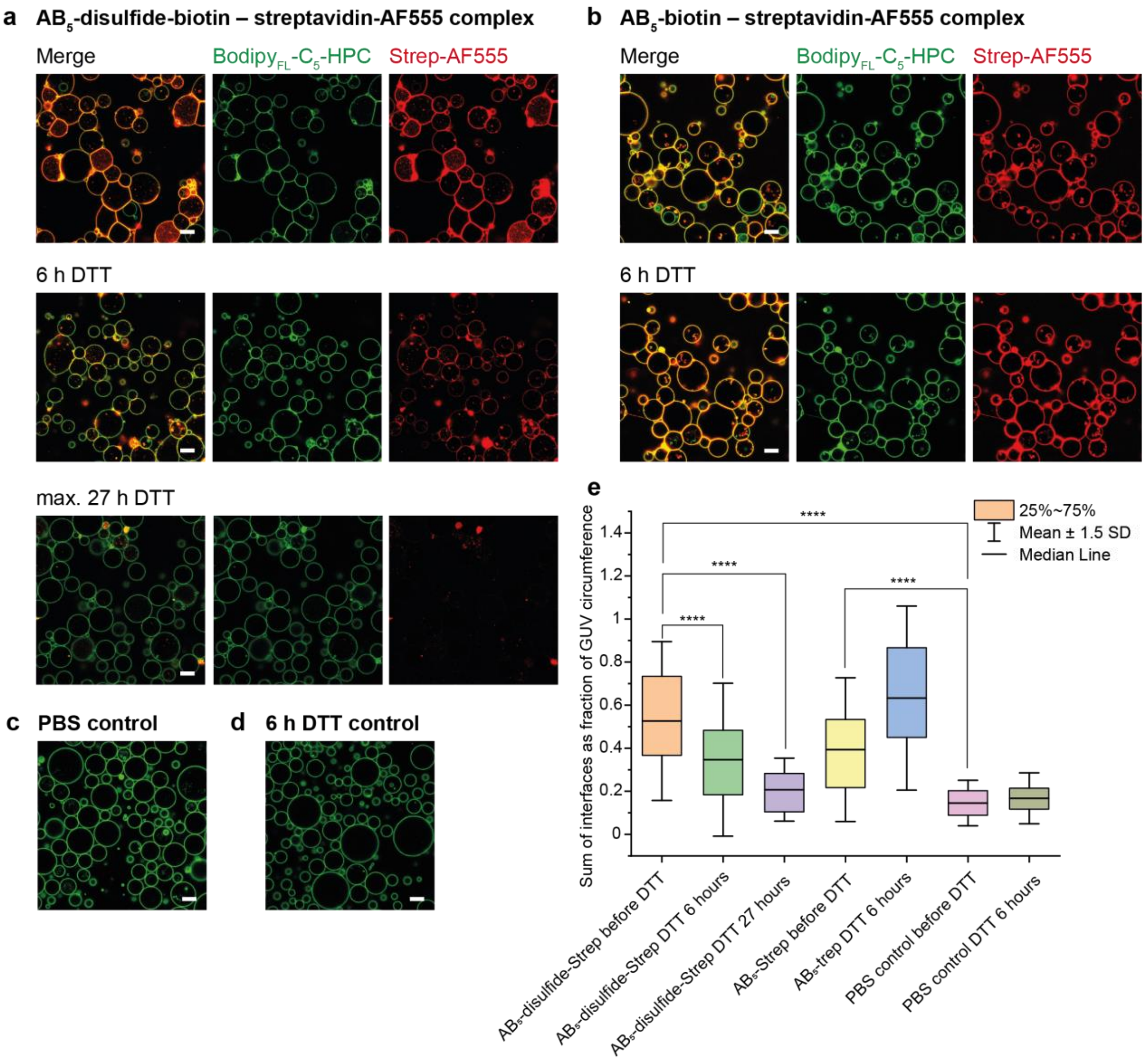

Supplementary Fig. S2: Crosslinking can be reversed by disassembling Strep-(AB_5)$_n$

Vesicles (1 mol% GM1 and 0.5 mol% $Bodipy_{FL}$-C_5-HPC (green)) (**a**) incubated with 120 nM AB_5-disulfide-biotin – streptavidin-AF555 (red) complex for 23 h showed vesicle crosslinking. A decrease of red fluorescence after 6 h treatment with 0.5 M DTT indicates cleavage of the disulfide bridge resulting in release of the two CTBs from the fluorescent streptavidin-biotin complex. A complete reversion of crosslinking was observed after incubation for 27 h. (**b**) Vesicles crosslinked by 120 nM of the previously introduced AB_5-biotin – streptavidin-AF555 complex overnight continued to increase their degree of crosslinking during the 6 h treatment with DTT. Control vesicles incubated with (**c**) PBS for 26 h showed limited contact between interfaces and were not influenced by (**d**) 6 h treatment with DTT. Scale bars are 10 µm. (**e**) Quantification of degree of crosslinking, as represented by the sum of vesicle interfaces as a fraction of GUV circumference. Data analysis was performed on multiple images (n = 3-5) corresponding to each condition displayed in panels (**a**)-(**d**).

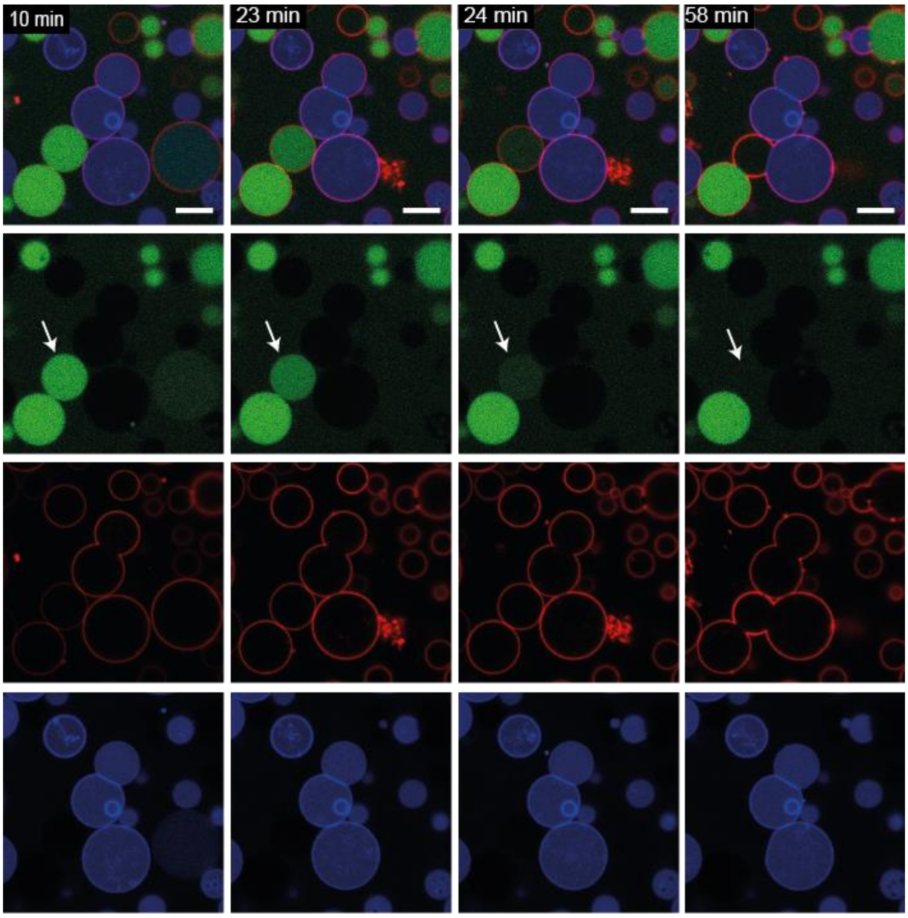

Supplementary Fig. S3: **Leakage of dextran filled vesicles**

Time-series captured in 1 min intervals of two slightly deflated vesicle populations (5 mol% GM1 without membrane staining) filled with either dextran-AF488 (green) or dextran-647 (blue) incubated with 200 nM AB_5-biotin – streptavidin-AF555 (red) complex. While the vesicles started to crosslink over time the indicated GUV (white arrow) started to leak, indicated by a gradual decrease of the fluorescence intensity within the vesicle until it reached the background level (58 min). Leakage had no major influence on the appearance of the vesicle and the deformation after 58 min can most probably be attributed to the crosslinking with the adjacent vesicles. Scale bars are 10 µm. For full time series see Supplementary Video SV6.

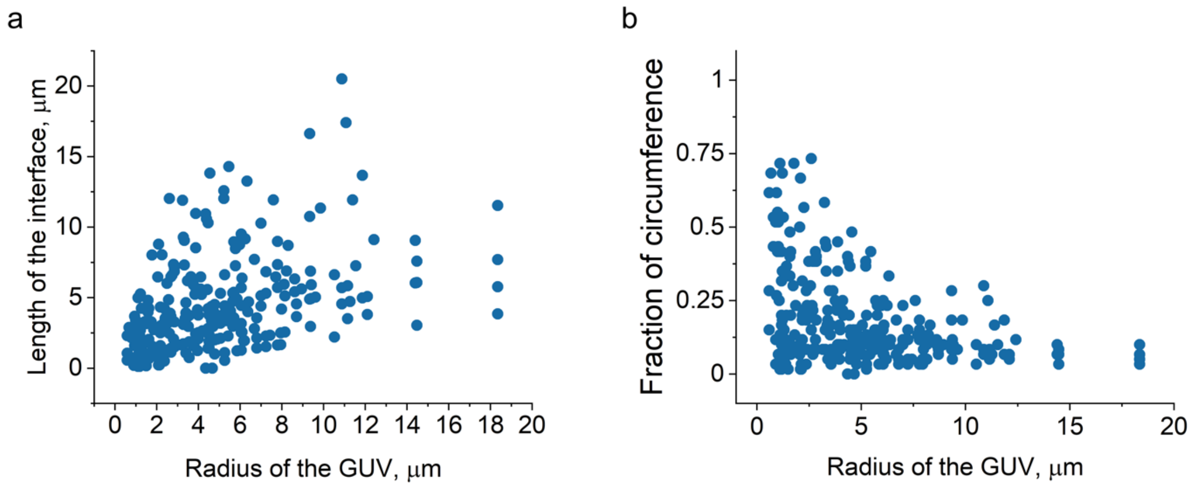

Supplementary Fig. S4: Length of hemifusion diaphragms between vesicles

Lengths of the interfaces in 2D images of the GUVs' cross-sections were measured as described on page 25 (n = 349; composition: DOPC/cholesterol/GM1 – 65/30/5, 200 nM of Strep-$(AB_5)_n$). All measurements were made using 2D images recorded in one focal plane, therefore some interfaces are not necessarily in the GUV's equatorial plane. **(a)** The plot illustrates the relationship of lengths of interfaces versus the radii of the GUVs **(b)** The fraction of GUV circumference that is formed of HDs versus the GUV radii.

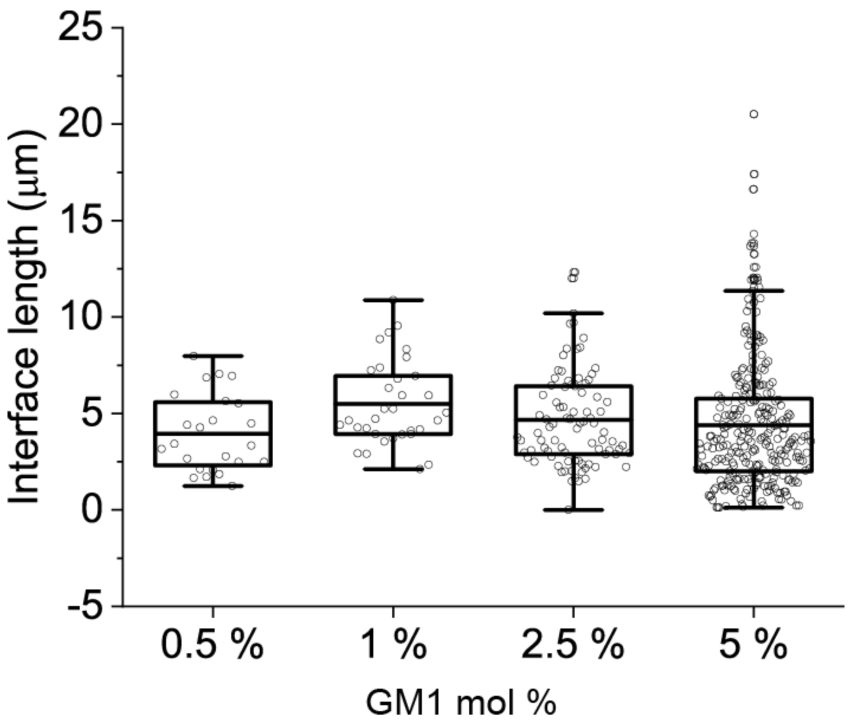

Supplementary Fig. S5 Sizes of the Strep-$(AB_5)_n$ induced interfaces, formed in GUVs with different GM1 percentage

200 nM Strep-$(AB_5)_n$ was used in all experiments. The lengths of the interfaces at equilibrium (when the interface stopped growing, and no fusion or explosion was observed) were measured using 2D images recorded in one focal plane as described on page 25. In cases which GUVs that constitute the interface undergo fusion or explosion, interface length was measured directly before the fusion or explosion of the GUVs. Horizontal lines depict mean values, boxes – 10-90%, whiskers – standard deviation. All data points are shown. Numbers of interfaces measured: 0.5% - 24, 1% - 33, 2.5% - 87, 5% - 277.

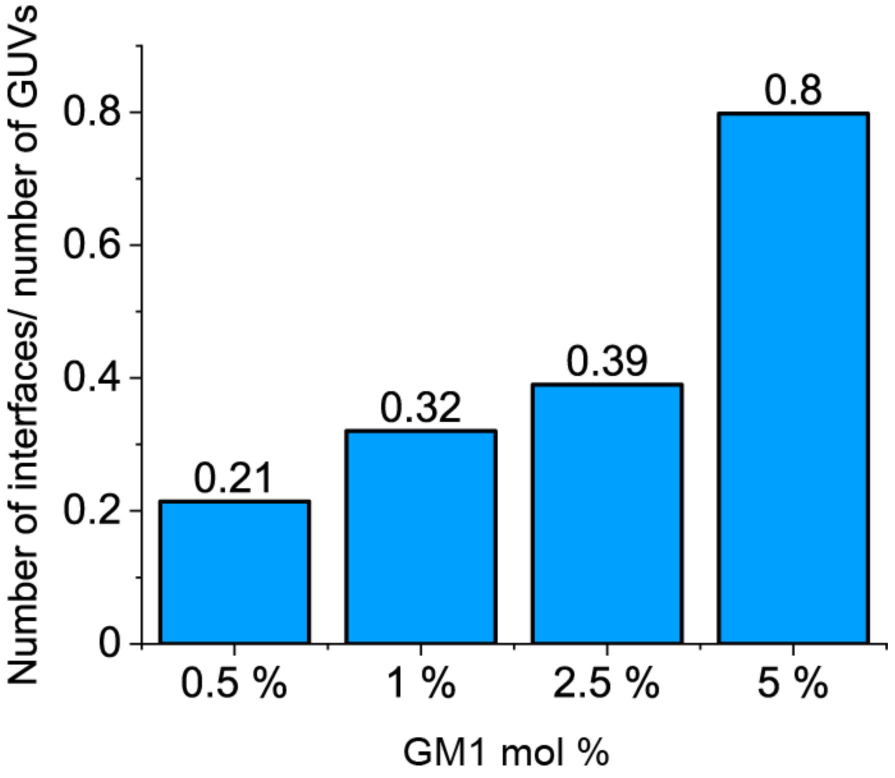

Supplementary Fig. S6: Average number of interfaces per GUV in presence of 200 nM Strep-$(AB_5)_n$

GUVs observed: 0.5% - 112, 1% - 103, 2.5% - 223, 5% - 347. The GUV density was kept equal in each experiment.

Supplementary Video Captions

Supplementary Video SV1: Biotinylated AB_5 protein induces membrane tubules

400 nM biotinylated AB_5 protein was added to vesicles functionalised with 1 mol% GM1 and 0.5 mol% $Bodipy_{FL}$-C_5-HPC (green) and resulted in mobile membrane tubules.

Supplementary Video SV2: Tubules distant from crosslinked interfaces can be induced by Strep-$(AB_5)_n$, related to Supplementary Fig. S1

Vesicles functionalized with 1 mol% GM1 and 0.5 mol% $Bodipy_{FL}$-C_5-HPC (green) were crosslinked with 120 nM AB_5-biotin – streptavidin-AF555 (red). White arrows point towards elongated interfaces while purple arrows indicate regions with tubules in the merge channel.

Supplementary Video SV3: Hemifusion, fusion, or vesicle rupture can be induced by Strep-$(AB_5)_n$, related to Fig. 3.

Two vesicle populations containing 2.5 mol% GM1 and either no membrane dye or 0.5 mol% DHPE-TxRed (red) were incubated with 100 nM AB_5-biotin – streptavidin-AF488 (green) complex. Yellow arrows in the red channel indicate hemifusion by the transfer of fluorescently labelled lipids between vesicles. The turquois arrow in the merge channel indicates a possible hemifusion diaphragm from which the complex becomes excluded which first increases in size until it subsequently decreases shortly before fusion illustrated by white arrows at the distal ends of the interface. The white circle in all channels indicates the complete fusion of two vesicles. Blue arrows in the merge channel point illustrate mobile domains of protein exclusion within one interface. The pink arrow in the merge channel points towards a vesicle which ruptures during the course of the time-lapse.

Supplementary Video SV4: Content mixing as a result of fusion induced by Strep-$(AB_5)_n$, related to Fig. 4.

Two vesicle populations (5 mol% GM1 without membrane staining) filled with either dextran-AF488 (green) or dextran-647 (blue) were incubated with 200 nM AB_5-biotin – streptavidin-AF555 (red) complex. White circles in all channels indicate the fusion of two vesicles inscribed as fusion event 1 or 2. The yellow arrow in the blue channel points towards a vesicle with a slight increase of blue fluorescence even before the first fusion event, which then increases after fusion event 1 (light orange arrow) and even further after fusion event 2 (orange arrow), resulting in a turquoise appearing vesicle. Turquoise arrows in the red channel indicate the exclusion of the complex from interfaces between the vesicles i, ii, and iii, which did not result in content mixing.

Supplementary Video SV5: Vesicle fusion does not necessarily follow nor require extensive vesicle crosslinking with complex exclusion, related to Fig. 5.

Two vesicle populations containing 5 mol% GM1 and either no membrane dye or 0.5 mol% DOPE-Atto488 (green) were incubated with 200 nM AB_5-biotin – streptavidin-AF555 (red) complex. Purple arrows in the merge channel illustrate the rupture of vesicles during the course of the time-lapse. Turquois arrows in the red channel indicate interfaces from which the complex became excluded. Yellow arrows in the green channel point towards vesicles which demonstrate transfer of fluorescently labelled lipids. White circles in all channels indicate the fusion of two vesicles during fusion event 1 and 2.

Supplementary Video SV6: Content leakage of dextran filled vesicles, related to Supplementary Fig. S3

Two vesicles populations (5 mol% GM1 without membrane staining) filled with either dextran-AF488 (green) or dextran-647 (blue) were incubated with 200 nM AB_5-biotin – streptavidin-AF555 (red) complex. The white arrow in the merge channel points towards a vesicle which loses its dextran-AF488 content during the course of the time-lapse. The pink arrow in the merge channel points towards a rupturing vesicle.

Supplementary Video SV7: Vesicle rupturing induced by Strep-$(AB_5)_n$

Two vesicle populations containing 2.5 mol% GM1 and either no membrane dye or 0.5 mol% DHPE-TxRed (red) were incubated with 100 nM AB_5-biotin – streptavidin-AF488 (green) complex. Rupture of vesicles started after approximately 60 min and resulted in remains of lipid debris.

Supplementary Video SV8: Hemifusion of liquid-ordered phase vesicles by Strep-$(AB_5)_n$

GUVs were constituted from lipid bilayers with a rigid liquid-ordered (Lo) phase (5 mol% GM1, 0.5 mol% Atto 647N-DOPE). When 20 µl Lo GUVs was incubated with 200 nM Strep-$(AB5)_n$ tubular invaginations were not visible. Lo GUVs were still observed to undergo crosslinking and HD formation, but no fusion.

Supplementary Video SV9: GUV control experiment in the absence of Strep-$(AB_5)_n$

Two vesicle populations containing 5 mol% GM1 and either no membrane dye or 0.5 mol% DHPE-TxRed (red) observed over 2 hours.

Experimental Methods

Materials

1,2-dioleoyl-*sn*-glycero-3-phosphocholine (DOPC), cholesterol, sphingomyelin and GM1 ganglioside from ovine brain were purchased from Avanti Polar Lipids; Cholera Toxin Subunit B (recombinant) Alexa Fluor 488 conjugate, Streptavidin Alexa Fluor 488 and 555 conjugates, Bodipy$_{FL}$ C_5-HPC, Texas Red 1,2-dihexadecanoyl-*sn*-glycero-3-phosphaethanolamine (DHPE-TxRed), Dextran Alexa Fluor 488 3,000 MW and Dextran Alexa Fluor 647 10,000 MW from Life Technologies; and ATTO 488 labelled 1,2-dioleoyl-***sn***-glycero-3-phosphoethanolamine (DOPE-Atto488) from ATTO-TEC. ATTO 647N labelled 1,2-dioleoyl-***sn***-glycero-3- phosphoethanolamine (DOPE-Atto647N), 1,4-dithiothreitol (DTT) and β-Casein were obtained from Sigma-Aldrich. All other reagents were from Sigma-Aldrich, Fisher Scientific, Melford Laboratories and VWR International. Commercial reagents were used without purification, unless otherwise stated.

Gene design for MBP-A2/CTB(T1A) AB_5 construct

Plasmid pSAB2.1 (Supplementary Fig. S7) was based on a synthetic gene ordered from Genscript for co-expression of maltose-binding protein-A2 and a T1A mutant of CTB (El Tor strain) subcloned into pMalp5x between the NdeI and PstI sites. pSAB2.1 includes a tobacco etch virus (TEV) protease recognition sequence followed by a triglycine linker and the El Tor cholera toxin A2 domain (residues 200-240). This is followed by a ribosome binding site, an *E. coli* heat labile toxin IIb periplasmic targeting sequence and the El Tor CTB sequence encoding a T1A mutation (out of frame with the MBP-A2 sequence to allow polycistronic expression of the genes). pTRBAB5-G1S was prepared from pSAB2.1 by QuikChange site-directed mutagenesis to change the ggtggcggt sequence encoding the triglycine linker to agtggcggt encoding SerGlyGly.

```
                                                  *agt*
aggatttcacatatgggatccgaaaacctgtactttcagggtggcggtgatgaaaaaacc
 R  I  S  H  M  G  S  E  N  L  Y  F  Q  G  G  G  D  E  K  T
caaagtcatggtgtaaaattccttgacgaataccaatctaaagttaaaagacaaatattt
 Q  S  H  G  V  K  F  L  D  E  Y  Q  S  K  V  K  R  Q  I  F
tcaggctatcaatctgatattgatacacataatagaattaaggatgaattatgacctcgag
 S  G  Y  Q  S  D  I  D  T  H  N  R  I  K  D  E  L  -
gtgaattcacgagcaattgaccaacaaggaccatagattatgagctttaagaaaattatc
                                          M  S  F  K  K  I  I
aaggcatttgttatcatggctgctttggtatctgttcaggcgcatgcagctcctcaaaat
 K  A  F  V  I  M  A  A  L  V  S  V  Q  A  H  A  A  P  Q  N
attactgatttgtgcgcagaataccacaacacacaaatatatacgctaaatgataagatc
 I  T  D  L  C  A  E  Y  H  N  T  Q  I  Y  T  L  N  D  K  I
ttttcgtatacagaatcgctagcgggaaaaagagagatggctatcattacttttaagaat
 F  S  Y  T  E  S  L  A  G  K  R  E  M  A  I  I  T  F  K  N
ggtgcaattttcaagtagaggtaccaggtagtcaacatatagattcacaaaaaaagcg
 G  A  I  F  Q  V  E  V  P  G  S  Q  H  I  D  S  Q  K  K  A
attgaaaggatgaaggataccctgaggattgcatatcttactgaagctaaagtcgaaaag
 I  E  R  M  K  D  T  L  R  I  A  Y  L  T  E  A  K  V  E  K
ttatgtgtatggaataataaaacgcctcatgcgatcgccgcaattagtatggcaaactaa
 L  C  V  W  N  N  K  T  P  H  A  I  A  A  I  S  M  A  N  -
gttttccctgcag
```

Supplementary Fig. S7. Sequence of the pSAB2.1 plasmid

Showing the last few residues of the pMalp5x sequence encoding a factor X cleavage sequence (blue); TEV protease sequence (yellow); GGG linker (pink) that was mutated to agt encoding a SGG linker at the position shown above the main sequence to make pTRBAB5-G1S for introduction of a serine residue at the N-terminus of the TEV-cleaved A2 peptide; the El Tor cholera A2 sequence (green); E. coli heat labile toxin IIb leader sequence (grey) and CTB(T1A) sequence (red).

Overexpression of MBP-AB$_5$ proteins from *E.coli*

A frozen glycerol stock of E. *coli* BL21 Gold cells harbouring the pSAB2.1/pTRBAB5-G1S plasmid was used to inoculate LB media (5 mL, 100 μg/mL ampicillin). The starter culture was incubated at 37 °C for 18 h before 3 ml was added to LB media (1 L, 100 μg/mL ampicillin), which was then grown at 37 °C. Protein overexpression was induced with IPTG (1 ml, 500 mM) once the OD_{600} had reached ~ 0.6. Incubation was continued for 20 h at 30 °C before the cells were isolated by centrifugation (10,000 × *g*, 10 min), the cell pellet was discarded and the supernatant retained.

The supernatant was filtered (0.8 μm filter, Sartorius Minisart) and passed down an amylose column (Bio-Rad) to capture MBP-AB$_5$ and uncomplexed MBP-CTA$_2$ proteins. The column was washed with PBS (5 × CV) and the proteins eluted with PBS buffer supplemented with

10 mM maltose (5 × CV). Protein-containing fractions were identified by the Bradford colorimetric assay and applied to a Ni-NTA sepharose (Qiagen) affinity column to isolate the MBP-AB_5 complex. Nickel affinity purification: The column was washed sequentially with PBS (5 × CV) and PBS supplemented with 25 mM imidazole (5 × CV) before the protein was eluted with PBS containing 500 mM imidazole (5 × CV).

The protein solution was concentrated to a total volume of 1 mL by centrifugal concentration at 4 °C before it was extensively dialysed into PBS at 4 °C. The purity of the isolated protein was determined by SDS-PAGE and the concentration determined by UV spectrometry at 280 nm using a theoretical molar extinction coefficient of 128745 M^{-1} cm^{-1}.

Cleavage of MBP-A2(G1S)/CTB AB_5 protein with Tobacco Etch Virus (TEV) protease

MBP-A2(G1S)/CTB AB_5 (44 μM, 600 μL) was incubated in the presence of MBP-TEV protease (4 μM, 100 μL) at r.t. for 1-2 hours with gentle agitation – the reaction was monitored by LCMS until only the cleaved MBP could be observed. The reaction mixture was immediately loaded onto a Ni-NTA column pre-equilibrated with PBS. It was important not to leave the cleavage reaction longer than necessary or non-specific proteolysis was sometimes observed. The column was washed with PBS (5 × CV) to remove cleaved MBP and MBP-TEV before the cleaved-protein was eluted with PBS supplemented with 300 mM imidazole (pH 7.4) (5 × CV). Protein-containing fractions were combined, centrifuged at 4 ºC and dialysed extensively in PBS at 4 ºC. The protein was characterised by ES-MS (Supplementary Figure S5) and SDS-PAGE (Supplementary Figure S6).

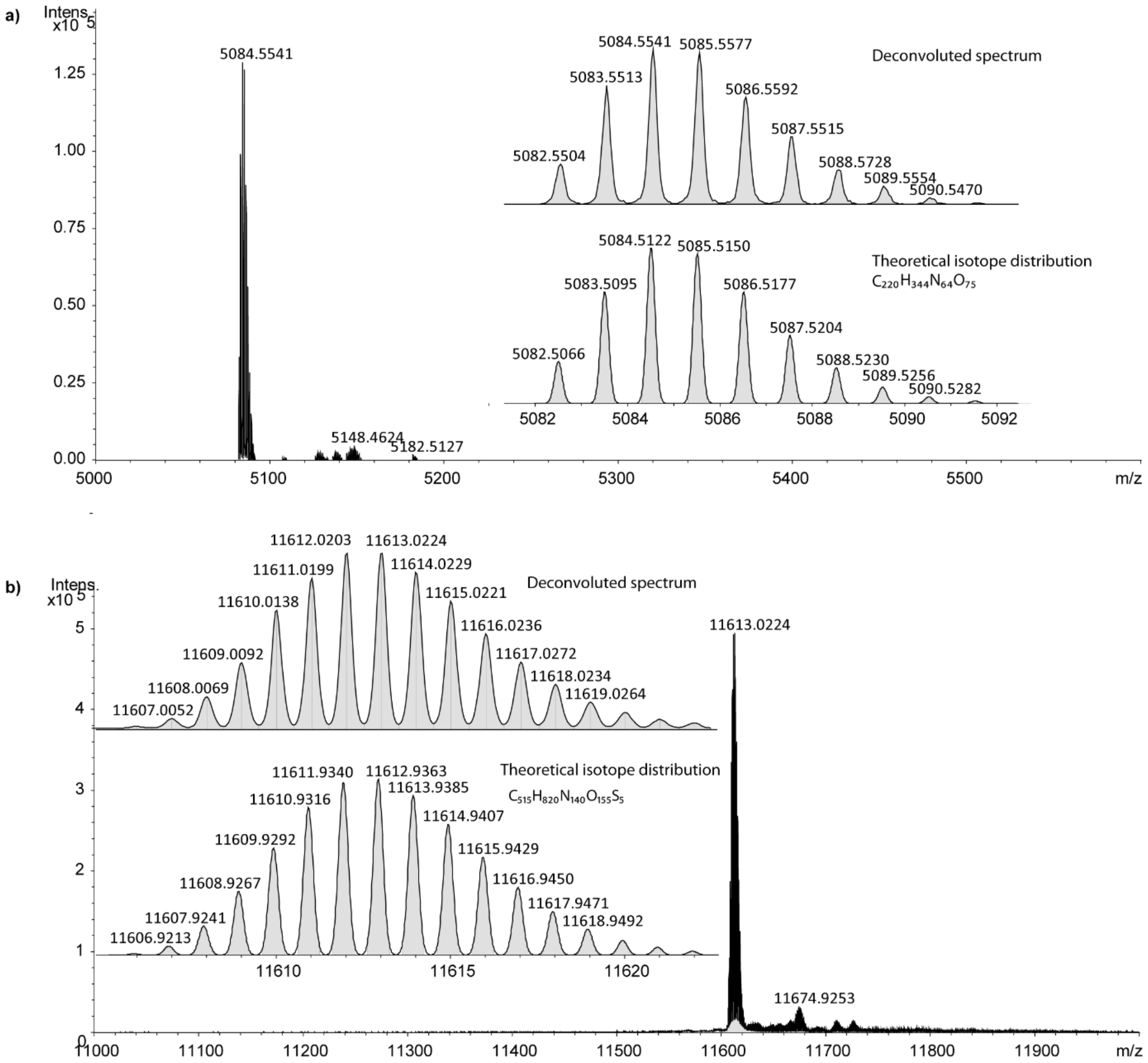

Supplementary Fig. S8. LC-MS of A2/CTB AB_5 protein derived from pTRBAB5-G1S.

Electrospray mass spectra recorded on a Bruker Daltonics MicroTOF instrument for a) the A2-peptide and b) the B-subunit following TEV cleavage and separation of the A2- and B-subunits.

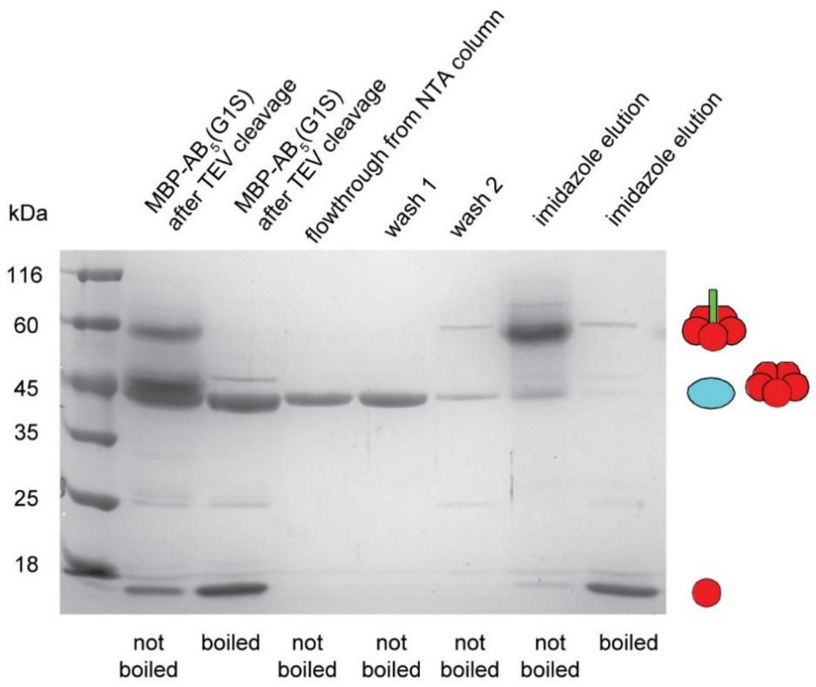

Supplementary Fig. S9. Purification of A2/CTB AB_5 on a Nickel-NTA resin.

Following TEV-cleavage, unboiled samples of the AB_5 species have an apparent size of just under 60 kDa. The band disappears on boiling as the AB_5 species dissociates into the A2 peptide (not seen) and B-protomer at ca. 12 kDa leaving the MBP protein at ca 45 kDa. Following washing of the column, the AB_5 protein is eluted with imidazole.

Typical procedure for periodate oxidation of A2(G1S)/CTB AB_5 protein

CTA2(G1S)/CTB AB_5 protein (35-45 μM) in PBS was incubated at room temperature with methionine (10 molar equivalents) and $NaIO_4$ (5 molar equivalents). Once LCMS analysis showed complete oxidation of the A2 peptide (typically 5-30 mins), the oxidised protein, AB_5-CTA2(G1S)ox protein was separated from excess periodate using a mini-trap GD-25 column and used immediately in the oxime ligation reaction.

Oxime ligation with Biotin-1

AB_5-CTA2(G1S)ox (15 μM), aniline (100 mM) and **Biotin-1** (EZ-Link™ Alkoxyamine-PEG4-Biotin, ThermoFisher; 250 mM stock solution in DMSO diluted to final concentration of 1.5 mM in the reaction mixture) were incubated at r.t. overnight. The reaction mixture was loaded onto a Ni affinity column (Qiagen, 10 ml) equilibrated in PBS. The column was washed with PBS (5 × CV) before the protein was eluted with PBS containing 300 mM imidazole. Protein-containing fractions were identified by the Bradford colorimetric assay,

concentrated to a total volume of 1 mL by centrifugal ultrafiltration at 4 °C and dialysed extensively against PBS at 4 °C. The protein was characterised by ES-MS (Supplementary Figure S7).

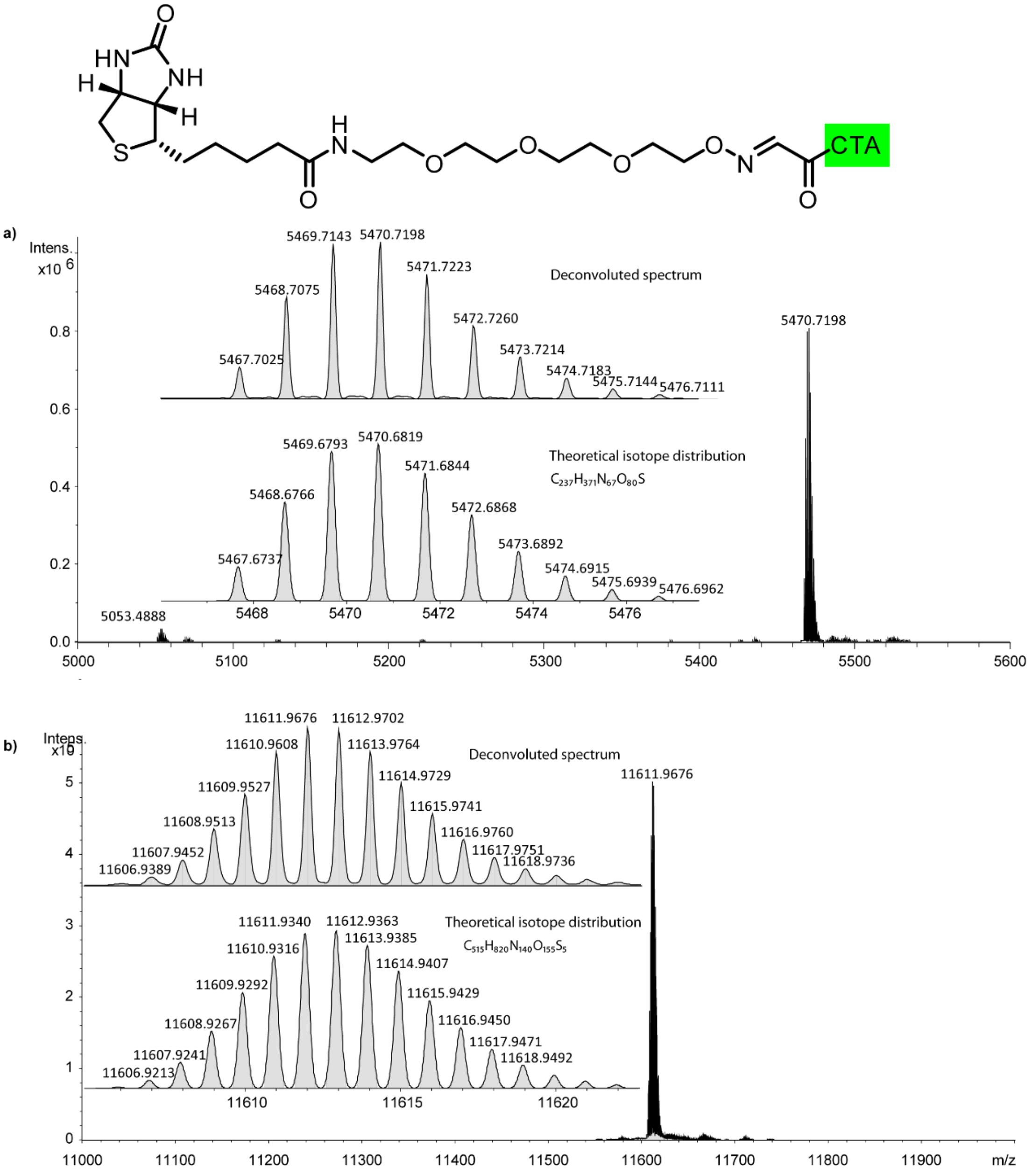

Supplementary Fig. S10. LC-MS of A2 peptide following oxime ligation.

Deconvoluted electrospray mass spectrum for a) the oxime of Biotin-1 and CTA2(G1S)ox and b) the unmodified B-subunit. The small peak at 5053.4888 corresponds to the mass of the oxidised protein CTA2(G1S)ox, but is more likely an unreactive aminal formed upon reaction with an adjacent amide nitrogen. High resolution electrospray mass spectrometry (ES+) was performed on a Bruker Daltonics MicroTOF instrument.

Synthesis of a disulfide-linked biotin oxyamine reagent (Biotin-2)

A Boc-protected derivative of **Biotin-2** (Supplementary figure S8) was synthesised from commercially-available N-bromopropylphthalimide **3** (Sigma-Aldrich), Boc-hydroxylamine **4** (Sigma-Aldrich) and EZ-link NHS-SS-biotin **6** (Thermo-Fisher). Oxyamine **4** was alkylated with bromopropylphthalimide **3** under reflux to give Boc-protected phthalimide **5** in 41% yield. Selective hydrazinolysis of the phthalimide group, and acylation with biotin derivative **6** in the presence of triethylamine formed Boc-protected oxyamine **7** in 54% yield. The reagent was stored in this form and deprotected with trifluoroacetic acid immediately before coupling to the oxidised protein.

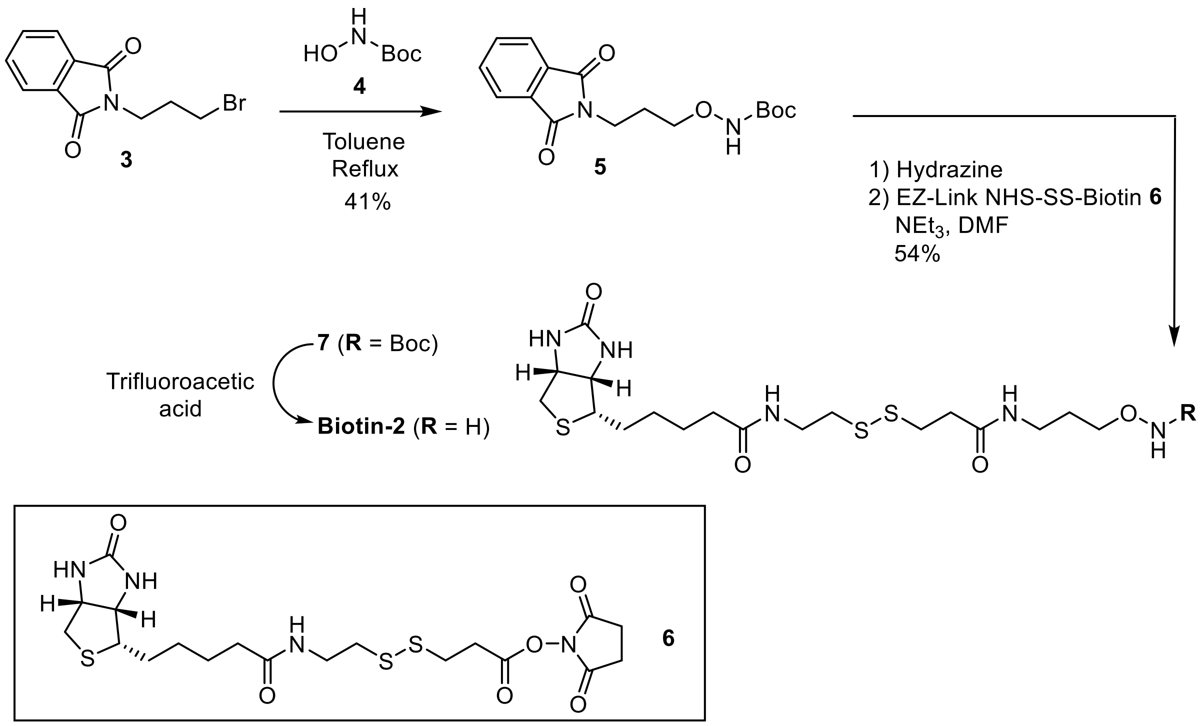

Supplementary Fig. S11. Synthesis of Biotin-2.

N-(3-(N-Boc-aminooxy)propyl)phthalimide 5[1]

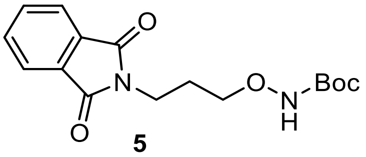

5

1,8-diazabicyclo[5.4.0]undec-7-ene (4.2 mL, 27.9 mmol) was added to a stirred solution of N-Boc-hydroxylamine (2.7 g, 20.5 mmol) and N-3-bromopropylphthalimide (5 g, 18.6 mmol) in toluene (50 mL). The reaction mixture was heated to reflux with stirring for 1 hour, cooled to r.t., concentrated and re-dissolved in DCM (100 mL). The solution was washed with 10% citric acid (2 × 100 mL) and brine (100 mL), dried over $MgSO_4$, filtered and concentrated. The crude product was purified using flash column chromatography (silica, 2:1 (v/v) hexane-EtOAc) to leave **5** as a white solid (2.6 g, 41%). R_F 0.35 (1:1 (v/v), hexane-EtOAc). **^{1}H NMR** (400 MHz, $CDCl_3$) δ = 7.87-7.78 (2H, m, ArH), 7.75-7.65 (2H, m, ArH), 7.30 (1H, bs, NH), 3.91 (2H, td, *J* 6.1 Hz, *J* 1.3 Hz, NCH_2), 3.81 (2H, td, *J* 7.0 Hz, *J* 1.3 Hz, OCH_2), 2.04-1.94 (2H, m, $CH_2C\underline{H_2}CH_2$), 2 x 1.46 (9H, s, C(CH3)$_3$). **^{13}C NMR** (100 MHz, $CDCl_3$) δ = 168.5 (CO), 157.0 (CO), 134.1 (ArC), 132.2 (ArC), 123.4 (ArC), 81.8 ($\underline{C}(CH)_3$), 74.1 ($O\underline{C}H_2$), 35.1 ($N\underline{C}H_2$), 28.3 (C($\underline{C}$H3)$_3$), 27.4 ($CH_2\underline{C}H_2CH_2$). **HRMS:** Found $[M+H]^+$ 321.1449, $C_{16}H_{21}N_2O_5$ requires 321.1445

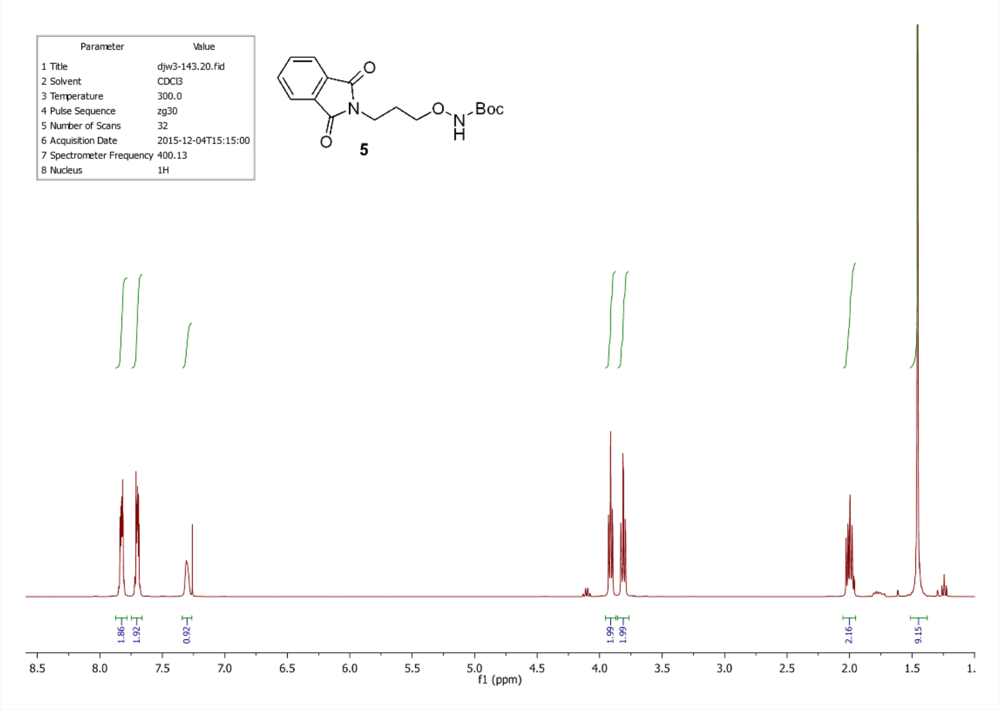

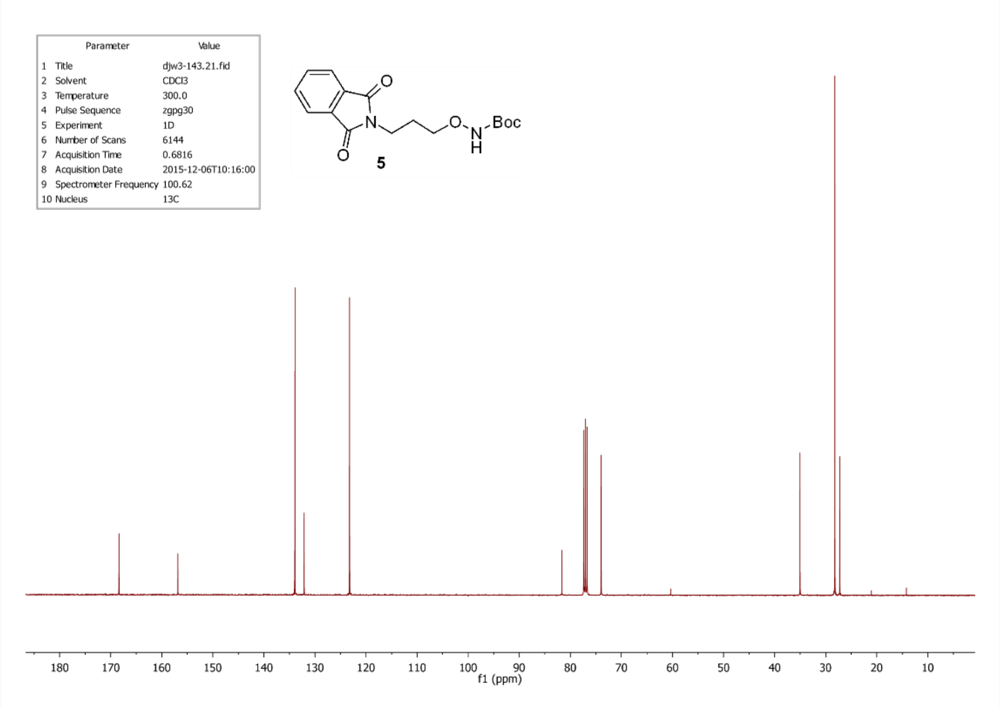

Supplementary Fig. S12. ^{1}H and ^{13}C NMR spectra for compound **5.**

Boc-protected biotin-SS-oxyamine 7

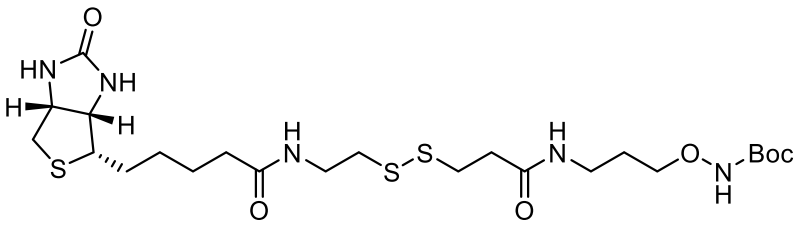

Hydrazine monohydrate (34 μL, 0.71 mmol) was added to a stirred solution of N-(3-(N-Boc-aminooxy)propyl)phthalimide **5** (200 mg, 0.59 mmol) dissolved in MeOH (5 mL) at r.t. The reaction mixture was heated to reflux and stirred for 1 hour before being concentrated and the resulting solid suspended in DCM. The suspension was stirred for 1 hour, filtered and washed extensively with DCM before the filtrate was collected and concentrated. The deprotected product (14 mg, 74 μmol) was dissolved in DMF before triethylamine (10.3 μL, 74 μmol) and EZ-link biotin-SS-NHS **6** (25 mg, 50 μmol) were added. The reaction mixture was stirred at r.t. overnight before being purified using HPLC and freeze-dried to give compound **7** as a lyophilisate (23 mg, 54%). **^{1}H NMR** (400 MHz, CD_3OD) δ = 4.53-4.45 (1H, m, $NHC\underline{H}CH/H'S$), 4.31 (1H, dd, *J* 7.9 Hz, *J* 4.5 Hz, $NHC\underline{H}CHS$), 3.84 (2H, t, *J* 6.1 Hz, $HNOCH_2$), 3.48 (2H, t, *J* 6.7 Hz, $NC\underline{H_2}CH_2SS$), 3.26-3.16 (1H, m, $NHCHC\underline{H}S$), 3.35-3.28 (2H, m, $NC\underline{H_2}CH_2CH_2$), 2.96 (2H, t, *J* 7.1 Hz, $SSC\underline{H_2}CH_2CO$), 2.96-2.91 (1H, m, $NHCHC\underline{H}/H'S$), 2.83 (2H, t, *J* 6.7 Hz, $NCH_2C\underline{H_2}SS$), 2.71 (1H, d, *J* 12.8 Hz, $NHCHCH/\underline{H}'S$), 2.61 (2H, t, *J* 7.1 Hz, $SSCH_2C\underline{H_2}CO$), 2.22 (2H, t, *J* 7.3 Hz, $CH_2CH_2CH_2C\underline{H_2}CO$), 1.85-1.73 (2H, m, $NCH_2CH_2CH_2$), 1.76-1.53 (4H, m, $C\underline{H_2}CH_2C\underline{H_2}CH_2CO$), 1.48 (9H, s, $C(C\underline{H_3})_3$), 1.49-1.42 (2H, m, $CH_2C\underline{H_2}CH_2CH_2CO$). **^{13}C NMR** (100 MHz, CD_3OD) δ = 176.2 (CO), 173.7 (CO), 166.1 (CO), 159.3 (CO), 82.1 ($\underline{C}(CH_3)_3$), 75.2 ($HNO\underline{C}H_2$), 63.4 ($NH\underline{C}HCHS$), 61.6 ($NH\underline{C}HCH/H'S$), 57.0 ($NHCH\underline{C}HS$), 41.0 ($NHCH\underline{C}H/H'S$), 39.5 ($N\underline{C}H_2CH_2SS$), 38.6 ($NCH_2\underline{C}H_2SS$), 37.7 ($N\underline{C}H_2CH_2CH_2$), 36.7 ($SSCH_2\underline{C}H_2CO$), 36.7 ($CH_2CH_2CH_2\underline{C}H_2CO$), 35.2 ($N\underline{C}H_2CH_2SS$), 29.7, 29.5 ($\underline{C}H_2\underline{C}H_2CH_2CH_2CO$), 28.7 ($NCH_2\underline{C}H_2CH_2$), 28.6 ($C(\underline{C}H_3)_3$), 26.8 ($CH_2\underline{C}H_2CH_2CH_2CO$). **HRMS:** Found $[M+H]^+$ 580.2314, $C_{23}H_{41}N_5O_6S_3$ requires 580.2292. **IR** (v_{max}/ cm^{-1}) 3290 (NH); 2930 (CH), 1698 (C=O).

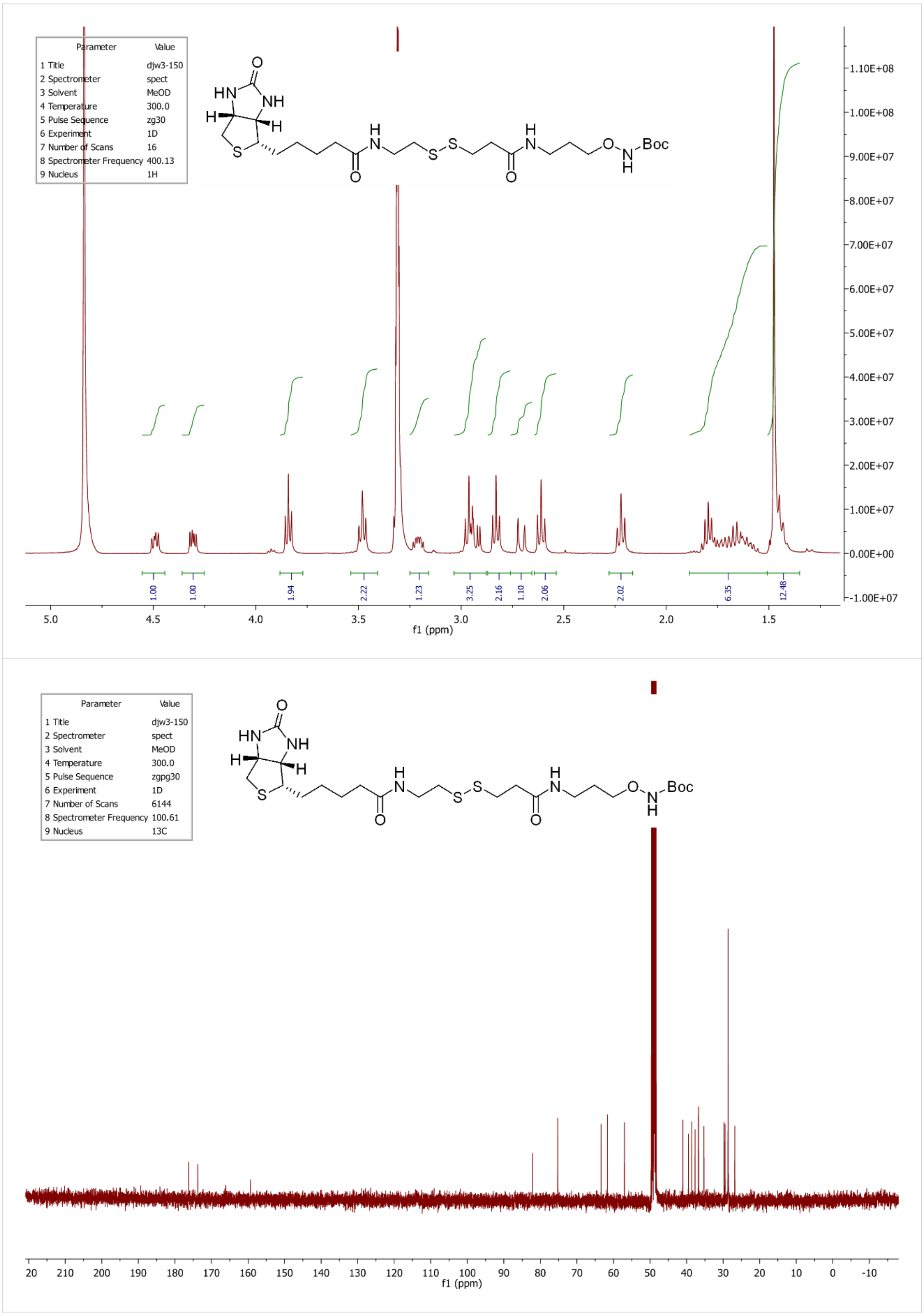

Supplementary Fig. S13. ^{1}H and ^{13}C NMR spectra for Boc-protected biotin-SS-oxyamine **7.**

Oxime ligation with disulfide-linked Biotin-2:

Boc-protected oxyamine **7** was treated with TFA for 30 mins at r.t. The reaction mixture was concentrated under a stream of N_2, the resulting residue dissolved in MeOH and evaporated again under a stream of N_2 - this was repeated 3 times to ensure all the TFA was removed. After the last evaporation, the residue was taken up into DMSO to give a 6 mM stock solution of **Biotin-2**.

AB_5-CTA2(G1S)ox (50 μM), aniline (100 mM) and disulfide-linked **Biotin-2** (1 mM) were incubated at r.t. overnight. The reaction mixture was loaded onto a Ni-NTA affinity column (Qiagen, 10 ml) equilibrated in PBS. The column was washed with PBS (5 × CV) before the protein was eluted with PBS containing 300 mM imidazole. Protein-containing fractions were identified by the Bradford colorimetric assay, concentrated to a total volume of 1 mL by centrifugal concentration at 4 °C and dialysed extensively against PBS at 4 °C.

Characterisation of the products by ES-MS (Supplementary Figure S11) demonstrated that the biotinylated AB_5 protein was successfully isolated (Supplementary Figure S11c); however, a smaller protein impurity (5389.5 Da) could also be observed by LCMS analyses. Its mass and isotope pattern were consistent with an aminooxy-linked byproduct (Supplementary Figure S11a,b) that would result from disulfide exchange between the biotin-SS-AB_5(G1S) product and the **Biotin-2** label. Other minor species present had masses consistent with an cyclic aminal (5053.4 Da) (while this is the same mass expected for the aldehyde, it is established that the aldehyde exists in its hydrated form in solution and observation of the "aldehyde" mass indicates formation of a cyclic hemiaminal involving an amide nitrogen from further along the chain[2]) and an oxime derived from hydroxylamine (5069.4 Da). As none of the byproducts of the reaction had a biotin group they would be unable to bind to streptavidin and therefor it was easier to remove them by size exclusion chromatography after complexation with streptavidin.

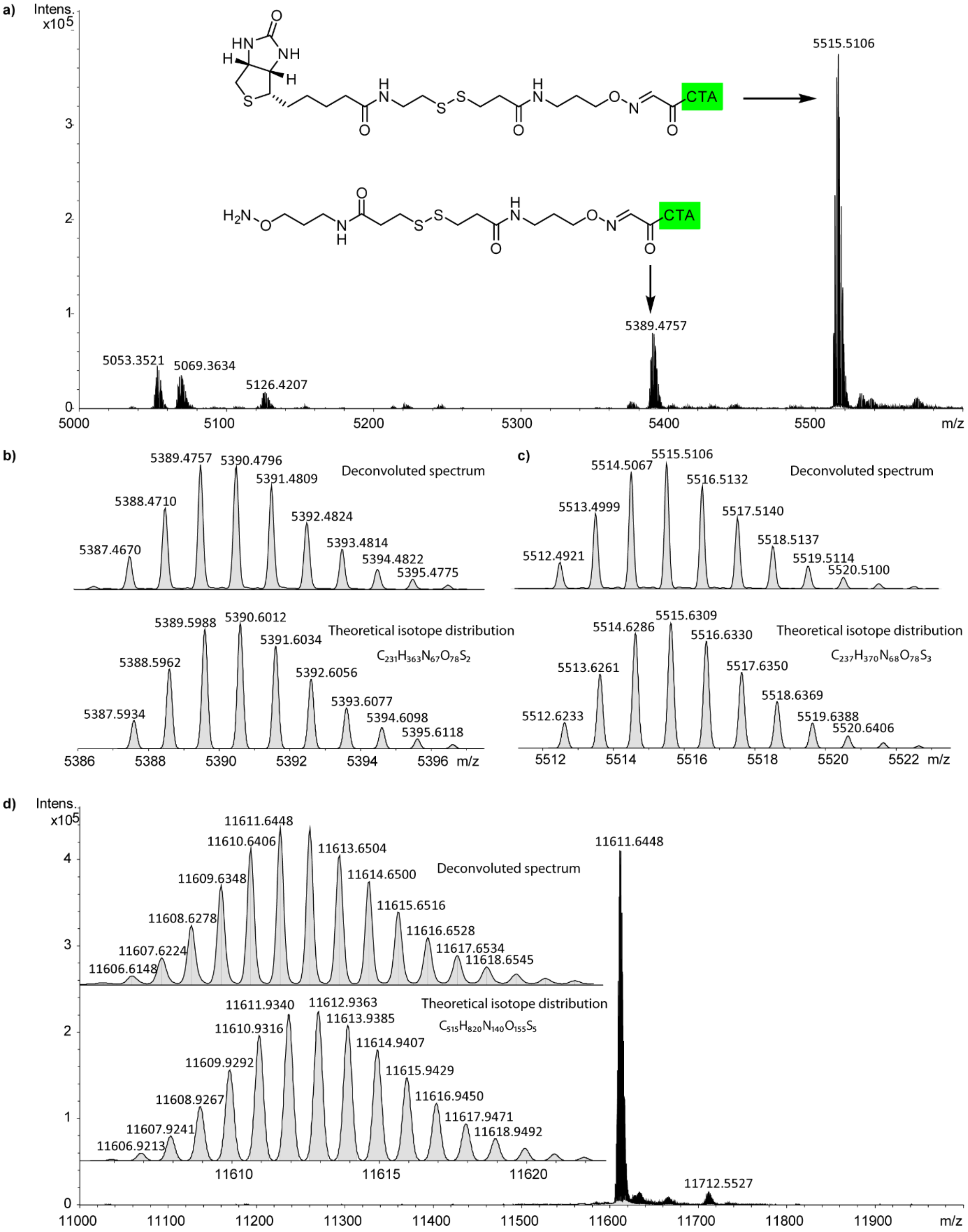

Supplementary Figure S14. LC-MS of A2 peptide following oxime ligation with **Biotin-2.** Deconvoluted electrospray mass spectrum for a) the oxime of Biotin-2 and CTA2(G1S)ox with expansions showing experimental and theoretical isotope patterns for b) a disulfide-exchange product and c) the desired biotinylated AB_5 protein product. d) Mass spectrum of the unmodified B-subunit with experimental and

simulated isotope patterns inset. High resolution electrospray mass spectrometry (ES+) was performed on a Bruker Daltonics MicroTOF instrument.

Complexation of Biotin-AB$_5$ variants with fluorescent streptavidin

Biotinylated AB$_5$ proteins (typically 15-60 µM in PBS) were mixed with an appropriate concentration of fluorescently-labelled streptavidin (Alexa Fluor® 488 or 555 streptavidin, Thermo Fisher). Size exclusion chromatography (SEC) on a Superose 6 or Superdex 200 (10/300) column (GE Healthcare) allowed separation of the complex from any excess, unbound AB$_5$ proteins (Supplementary figure S12).

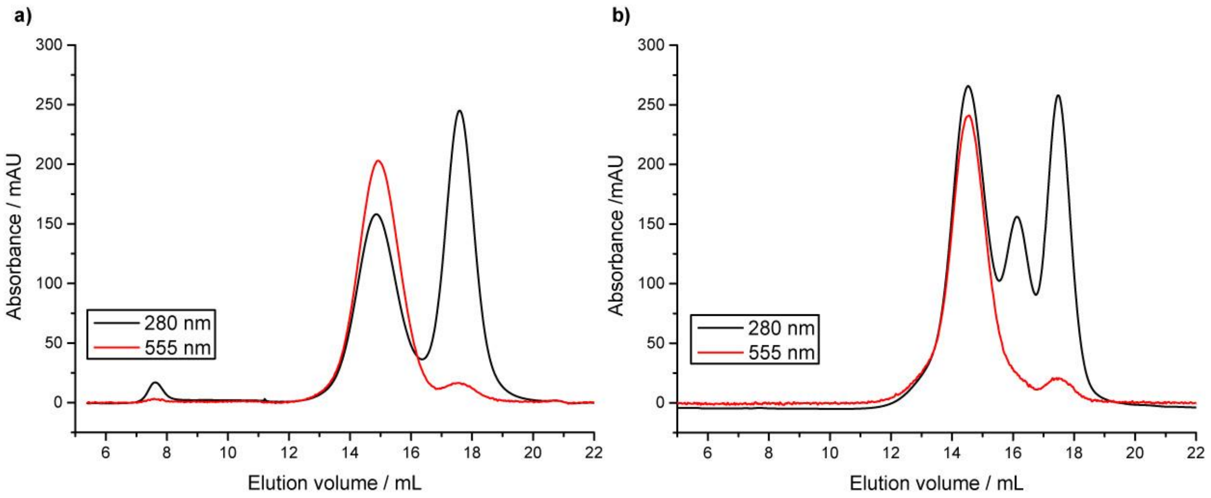

Supplementary Figure S15. Size exclusion chromatograms of Alexa-555-labelled streptavidin-AB$_5$ complexes.

Size exclusion chromatography on a Superose 6 (10/300) column (GE Healthcare) monitored by UV/vis absorbance at 280 nm to show all proteins and at 555 nm to show the streptavidin-containing species: a) chromatogram for the PEG-linked Biotin-1-AB$_5$ protein shows an additional peak at 18 mL corresponding to uncomplexed AB$_5$ protein; b) chromatogram for the disulfide-linked Biotin-2-AB$_5$ protein shows two additional peaks, presumably corresponding to uncomplexed AB$_5$ protein and a disulfide-linked AB$_5$ dimer.

Isothermal titration calorimetry (ITC)

ITC titrations were performed using a MicroCal ITC_{200} calorimeter (GE Healthcare) with a cell volume of 0.2028 mL and data processed and fitted in Origin using standard MicroCal software. Protein samples were dialysed into PBS (SnakeSkin® pleated dialysis tubing, Thermo Scientific, with 7000 MWCO) prior to analysis. Titrations typically comprised 20 injections of 2 µL at 2 minute intervals. Separate titrations of the ligand into buffer were used to subtract the heat of dilution from the integrated data prior to curve fitting.

Determination of complex stoichiometry using SEC-MALLS

Fifty microliters of protein mixture of streptavidin and biotin-AB_5 with a total concentration of 3 mg/ml was injected onto a WTC-MP030S5 column, equilibrated with PBS buffer pH 7.6 at a flow rate of 1 ml/min. Data were recorded using a DAWN 8+ multi-angle light scattering (LS) detector, an Optilab T-rEX differential refractive index (dRI) detector and UV absorbance (UV) detector (Wyatt Technology Corporation) and analysed using Astra6.2 (Wyatt Technology).

Since the molecular masses of the single proteins are very similar (streptavidin 66 kDa; biotin-AB5 63 kDa) determining the molecular mass of the streptavidin:biotin-AB_5 complex alone is not sufficient to identify the correct stoichiometry as 1:3/2:2 (255/258 kDa) and 1:2/2:1 (192/195 kDa) are indistinguishable. To determine the stoichiometry of the complex, we adjusted the method from Wen *et al*.[3] The molecular masses were first calculated using the dRI signal as concentration source, afterwards the molecular masses were determined using the UV signal as concentration source with various ratios of the UV extinction coefficients representing different complex stoichiometries. The best fit with the determined molecular mass *via* dRI provides the correct complex stoichiometry.

Retention time	dRI calculated molecular mass (kDa)	Assumed stoichiometry of streptavidin:biotin-AB5	ε (ml mg^{-1} cm^{-1})	UV calculated molecular mass (kDa)	Best fit	conclusion
7.1 min	247 ± 8.6	1:3	1.36	237.6 ± 8.3	yes	1:3 complex
		2:2	1.755	306.2 ± 10.7	no	
7.5 min	198 ± 3.3	1:2	1.509	192 ± 3.2	yes	1:2 complex
		2:1	2.018	258.1 ± 4.3	no	
9.4 min	63.3 ± 1.2	0:1	0.966	61.4 ± 1.2	yes	biotin-AB_5 in excess
		1:0	2.545	161.8 ± 3.1	no	

GUV preparation

The electroformation technique was used to prepare GUVs as previously described by Madl et al. (2017).[4] In brief, lipids dissolved in chloroform to a total concentration of 0.5 mg ml^{-1} were spread on indium-tin oxide (ITO)-coated slides followed by evaporation of the solvent. An alternating electric field with a field strength of 1 V/mm was applied at room temperature for 3 h in either ~276 mOsm L^{-1} or ~266 mOsm L^{-1} sucrose solution. The lipid preparations contained 30 mol% cholesterol, 0.5 mol% of membrane dye, and 68.5 mol% DOPC for 1 mol% GM1, 67 mol% DOPC for 2.5 mol% GM1, and 64.5 mol% DOPC for 5 mol% GM1, respectively. For high order and rigid only GUVs (known as liquid-ordered (Lo) GUVs), lipid preparation was made of 64.5 mol% sphingomyelin, 30 mol% cholesterol, 0.5 mol% membrane dye and 5 mol% GM1. Electroformation of Lo GUVs was made above phase transition temperature for all lipids.[5]

Observation chambers

Home-built observation chambers were prepared as described in Madl et al. (2017).[4] In short, 8 ×8 mm cloning cylinders were glued to coverslips to enable the possibility to add the toxin complex or other compounds. Unspecific adhesion of the vesicles to the glass was prevented by coating the chambers with 1mg/ml β-Casein in Tris buffer 30 min prior to the experiment. GUVs were sedimented in chambers filled with PBS (~278 mOsm) due to the higher density of sucrose and became slightly deflated when they were electroformated in the hypoosmotic

~266 mOsm L^{-1} sucrose solution. The toxin complex or other compounds were added to the GUVs in an equivalent volume of PBS to the volume of PBS and GUVs in sucrose solution to constitute an evenly distributed intermixture of a final indicated complex concentration. Only in Supplementary Fig. 2, the disulfide and conventional complex were added to PBS prior to the addition of the GUVs, as the reducing agent DTT was added subsequently.

Lipid mixing assay

Two different vesicle populations were used in order to identify the transfer of a fluorescently labelled lipid from one vesicle to the other indicating hemifusion. The green fluorescent fatty acid $Bodipy_{FL}$ C_5-HPC was not suited for this assay, as a transfer between vesicles was observed in the PBS control. Furthermore, to avoid fluorescence resonance energy transfer (FRET) of different fluorophores within one membrane, one vesicle population was unstained.

Content mixing assay

GUVs were electroformated as usual with dextran-AF488 or dextran-AF647 solved in sucrose solution (0.03 mg/ml). Accordingly, dextran was not only encapsulated within the vesicles but also present in the surrounding sucrose solution, yet the latter was significantly diluted by PBS within the chamber resulting in a weak background and a much stronger fluorescence signal within the liposomes.

Microscopic imaging

GUVs were imaged on a confocal microscope (Nikon Eclipse Ti-E inverted microscope equipped with a Nikon A1R confocal laser scanning system, 60x oil immersion objective, NA=1.49, 4 laser lines: 405 nm,488 nm, 561nm, 640 nm; Nikon Instruments). The software NIS-elements (Nikon) was used for image acquisition and analysis. To follow the events induced by the complex, several positions within one chamber were recorded with one image per minute for about 2 h.

Image processing

The quantitative image analysis was performed using Fiji.[6] Interfaces are two dimensional structures (areas). However, as they are usually not arranged parallel to the focal plane they appear as one dimensional structures (lines) in the fluorescence images. We therefore used the length of the apparent interface as a measure for interface size. The quantification of numbers and sizes of the GUVs and GUV interfaces (including the hemifusion diaphragms) for

Supplementary figures 4-6 was based on images that contained two colour channels: one colour channel containing the fluorescence signal from the membrane probe (so-called "membrane" channel) and one colour channel containing the fluorescence signal from the labelled CTB construct (so-called "construct" channel). The image processing was performed using the home-made Fiji macro based on our recently developed GUV analysis software (GUV-AP).[7] Briefly, GUVs were detected using the "membrane" channel (Supplementary figure S13). Furthermore, circular profiles of all GUVs were extracted in both colour channels. Interfaces were detected and analysed using the circular profiles of the GUVs extracted from the "construct" colour channel. The macro source code is available via GitHub repository at https://github.com/taras-sych/GUV-AP/releases.

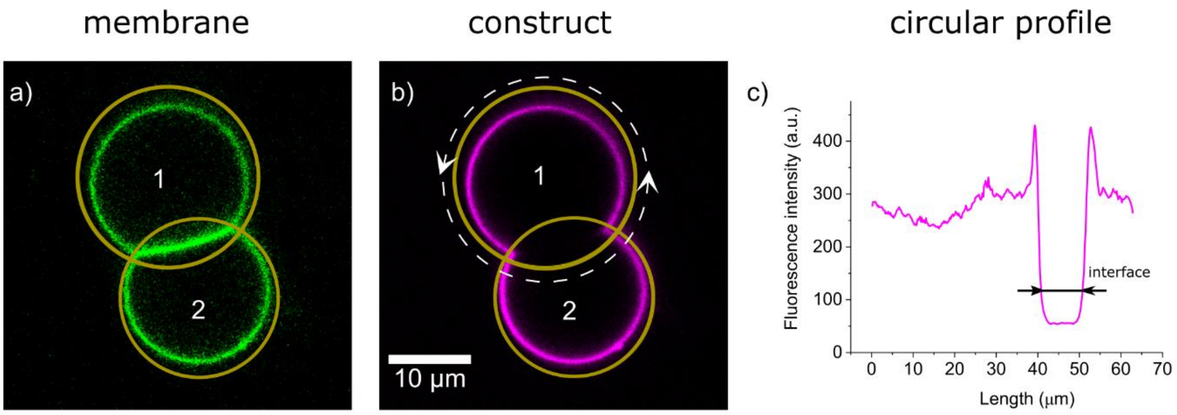

Supplementary Figure S16. Example of quantification of lengths of the interfaces.

As interfaces are usually not arranged parallel to the focal plane they appear as one dimensional structures (lines) in the fluorescence images. We therefore used the length of the apparent interface as a measure for interface size. The two colour image (containing "membrane" channel(a) and "construct" channel(b)) is used. GUVs (a, b – 1 and 2) are detected using "membrane" channel. For each GUV the circular profile is extracted. In (b) the dashed circular arrow indicates the circular profile of GUV1 which is depicted on the plot in (c). The interface (in this case also a hemifusion diaphragm) is detected using the circular profile (c) and measured.

References for Supporting Information

(1) de Graaf, A. J.; Mastrobattista, E.; Vermonden, T.; van Nostrum, C. F.; Rijkers, D. T. S.; Liskamp, R. M. J.; Hennink, W. E. Thermosensitive Peptide-Hybrid ABC Block Copolymers Obtained by ATRP: Synthesis, Self-Assembly, and Enzymatic Degradation. *Macromol.* **2012**, *45*, 842-851.

(2) Rose, K.; Chen, J.; Dragovic, M.; Zeng, W.; Jeannerat, D.; Kamalaprija, P.; Burger, U. New Cyclization Reaction at the Amino Terminus of Peptides and Proteins. *Bioconjug. Chem.* **1999**, *10*, 1038-1043.

(3) Wen, J.; Arakawa, T.; Philo, J. S. Size-Exclusion Chromatography with On-Line Light-Scattering, Absorbance, and Refractive Index Detectors for Studying Proteins and Their Interactions. *Anal. Biochem.* **1996**, *240*, 155-166.

(4) Madl, J.; Villringer, S.; Römer, W.: Delving into Lipid-Driven Endocytic Mechanisms Using Biomimetic Membranes. In *Chemical and Synthetic Approaches in Membrane Biology*; Shukla, A., Ed.; Humana Press: New York, NY, 2016; pp 17-23.

(5) Sych, T.; Mély, Y.; Römer, W. Lipid self-assembly and lectin-induced reorganization of the plasma membrane. *Phil. Trans. R. Soc. B* **2018**, *373*, 20170117.

(6) Schindelin, J.; Arganda-Carreras, I.; Frise, E.; Kaynig, V.; Longair, M.; Pietzsch, T.; Preibisch, S.; Rueden, C.; Saalfeld, S.; Schmid, B.; Tinevez, J.-Y.; White, D. J.; Hartenstein, V.; Eliceiri, K.; Tomancak, P.; Cardona, A. Fiji: an open-source platform for biological-image analysis. *Nature Methods* **2012**, *9*, 676-682.

(7) Sych, T.; Schubert, T.; Vauchelles, R.; Madl, J.; Omidvar, R.; Thuenauer, R.; Richert, L.; Mély, Y.; Römer, W. GUV-AP: multifunctional FIJI-based tool for quantitative image analysis of Giant Unilamellar Vesicles. *Bioinformatics* **2019**, *35*, 2340-2342.
